# Early warning of critical transitions in biodiversity from compositional disorder

**DOI:** 10.1002/ecy.1558

**Published:** 2016-11-03

**Authors:** C. Patrick Doncaster, Vasthi Alonso Chávez, Clément Viguier, Rong Wang, Enlou Zhang, Xuhui Dong, John A. Dearing, Peter G. Langdon, James G. Dyke

**Affiliations:** ^1^ Biological Sciences Institute for Life Sciences University of Southampton Southampton SO17 1BJ UK; ^2^ Institute for Complex Systems Simulation University of Southampton Southampton SO17 1BJ UK; ^3^ University of Nice Polytech Nice‐Sophia Sophia‐Antipolis Cedex 06903 France; ^4^ Geography and Environment University of Southampton Southampton SO17 1BJ UK; ^5^ State Key Laboratory of Lake Science and Environment Nanjing Institute of Geography and Limnology Chinese Academy of Sciences Nanjing 210008 China; ^6^ Aarhus Institute of Advanced Studies Høegh‐Guldbergs Gade 6B Aarhus C DK‐8000 Denmark

**Keywords:** critical slowing down, early warning signal, ecosystem canary, lake sediment, leading indicator, nestedness temperature, regime shift, tipping point

## Abstract

Global environmental change presents a clear need for improved leading indicators of critical transitions, especially those that can be generated from compositional data and that work in empirical cases. Ecological theory of community dynamics under environmental forcing predicts an early replacement of slowly replicating and weakly competitive “canary” species by slowly replicating but strongly competitive “keystone” species. Further forcing leads to the eventual collapse of the keystone species as they are replaced by weakly competitive but fast‐replicating “weedy” species in a critical transition to a significantly different state. We identify a diagnostic signal of these changes in the coefficients of a correlation between compositional disorder and biodiversity. Compositional disorder measures unpredictability in the composition of a community, while biodiversity measures the amount of species in the community. In a stochastic simulation, sequential correlations over time switch from positive to negative as keystones prevail over canaries, and back to positive with domination of weedy species. The model finds support in empirical tests on multi‐decadal time series of fossil diatom and chironomid communities from lakes in China. The characteristic switch from positive to negative correlation coefficients occurs for both communities up to three decades preceding a critical transition to a sustained alternate state. This signal is robust to unequal time increments that beset the identification of early‐warning signals from other metrics.

## Introduction

Much current research on living systems concerns methods of predicting distances to critical transitions, defined as the point at which a system becomes entrained into an inevitable future change of state. Analysis of how populations change over time has revealed statistical signals of an approaching critical transition such as slowing down, amplified variance and skew, and flickering between regimes (Lenton et al. 2008, Scheffer et al. 2009, 2012). In model systems, such metrics may provide generic early warnings of a nonlinear response with hysteresis (Carpenter and Brock 2006, Guttal and Jayaprakash 2008, Chisholm and Filotas 2009, Scheffer et al. 2009). Because they inform on the process of system failure and not on its mechanism, however, they are inherently vulnerable to false positives arising from reversible processes such as a run of poor seasonal conditions that may generate the same signals. Analyses of the drivers of critical transitions reveal other signals in community composition (Scheffer and van Nes 2007), or even an absence of signals (Hastings and Wysham 2010), and some have argued that rising variance may simply identify losses in system resilience (Kéfi et al. 2012). Almost all such analyses are model‐based studies, and few real‐world examples exist of complex ecosystems covering the multi‐decadal timescales over which ecosystems respond to drivers (Capon et al. 2015, cf. experimental data on 4 yr recovery from a manipulated critical transition: Carpenter et al. 2011, Seekell et al. 2012, Pace et al. 2013). Metric‐based measures of early warnings from “Quickest detection” methods (Carpenter et al. 2014) need a reasonable understanding of dynamic processes in the system in order to know the relevant measurement variables. Such understanding is often lacking in real‐world ecosystems. More work is required over multi‐decadal timescales to understand the processes and structural changes underlying critical transitions (Scheffer et al. 2012), and which classes of models best represent natural system dynamics (Hastings and Wysham 2010).

The aim of this paper is to demonstrate that changes in community structure can convey diagnostic information about an approaching critical transition. We present a population‐based early‐warning signal of a critical transition in real‐world lake ecosystems. We identify characteristic changes in the functional composition of lake algae (diatom) and zoobenthos (chironomid) communities that presage critical transitions under environmental forcing. Our central assumption is that interactions amongst species competing for similar resources within the lake sustain three functional categories of species: keystone species exhibiting slow self‐replication compensated by strong competitive ability; weedy species exhibiting fast self‐replication at the expense of poor competitive ability; canary species exhibiting both slow self‐replication and poor competitive ability. As environmental degradation impacts an ecosystem, keystones initially prevail through competitive dominance over others, resulting in the early demise of canary species. With continuing degradation affecting all species, however, the faster replicating weedy species eventually experience competitive release initiating their proliferation to higher carrying capacities than were possible under keystone regulation. The loss of keystones entrains the ecosystem into a critical transition to an alternate state characterized by the bloom of weedy species. The intrinsic vulnerability of canary species suggests a role for this functional group as a sensitive indicator of environmental forcing, yet it is often assumed that their presence or absence imparts no useful ecological information.

Time‐series of biodiversity present challenges for identifying functional categories. Although certain taxa are known to associate with different lake states, they have lake‐ and time‐specific ecological functions in the community. Higher‐level analysis seems also to present limited opportunities for interpreting changes in community structure. Biodiversity alone informs only on numbers of species not relative prevalence of functional categories. Here we show that we can make robust inferences about changes in functional structure by correlating biodiversity with compositional disorder, which measures the degree of unpredictability in composition of sequential samples.

Our objectives are to (1) analyze population dynamics of species that compete for similar resources; (2) build the dynamics into a stochastic simulation of a community under environmental forcing to generate predictions for structural changes in community composition; (3) test predictions on replicate paleo datasets of diatoms and chironomids (nonbiting midges) from lakes that have undergone critical transitions to significantly different states. The empirical availability of replicate critical transitions, and reference before‐after comparators, provide a rare opportunity to distinguish signals from stochastic events in a stable system (Boettiger and Hastings 2013). Metrics‐based leading indicators all require independent and equal time intervals to identify significant change from the null model, which is often impossible to obtain from lake sediments when combined with the need for high resolution data (Carstensen et al. 2013). Our analysis avoids this issue by testing only for a change in the alignment of two variables with each other. Consequently, it is unimpeded by sediment compaction or associated decreasing time resolution with core depth.

## Materials and Methods

### Conceptual model of species competing for similar resources

Here we develop a standard Lotka‐Volterra model of community composition under environmental forcing. This analysis provides the phenomenological basis for a stochastic agent‐based simulation, from which we derive an empirically testable metric for predicting an approaching critical transition. Both analysis and simulation use the simplest representations of density dependent exploitation, in order to predict generic responses to interspecific competition that can apply also to more community‐specific mechanistic models. They build on previous analyses by Nee and May (1992), Tilman (1994), Doncaster and Gustafsson (1999), and Doncaster (2009).

Let each species *i* in the community have an intrinsic lifetime fitness, *R*_*i*_ = *c*_*i*_/*d*_*i*_, where *c*_*i*_ and *d*_*i*_ are pre‐competition vital rates of individual birth and death. Species *i* receives impact α_*ij*_ on population growth from each species *j*, calibrated against the intraspecific impact α_*ii*_ = 1, and it delivers impact α_*ji*_ on *j* calibrated against its intraspecific impact α_*jj*_ = 1. The population dynamics of a well‐mixed community of *S* species are captured by a rate equation for change over continuous time in abundance *n*_*i*_ of each species *i*:

(1) $${\dot{n}}_{i}=c_{i}\left[h_{i}-\sum_{j=1}^{S}\alpha_{ij}\cdot n_{j}\right]\cdot n_{i}-d_{i}\cdot n_{i},$$

where *n*_*i*_ is a fraction of carrying capacity *k*_*i*_ = *h*_*i*_−*d*_*i*_/*c*_*i*_ before interspecific competition, and *h*_*i*_ is the intrinsic quality or amount of habitable environment for species *i* as a fraction of its pristine environment. The rate equations for a community of *S *=* *2 species achieve dynamic equilibrium abundance $n_{i}^{*}$ (*i *=* *1, 2) when ${\dot{n}}_{i}=0$, at:

(2) $$n_{i}^{*}=\left(k_{i}-\alpha_{ij}\cdot k_{j}\right)/\;\left(1-\alpha_{ij}\alpha_{ji}\right)\;\text{for}\;n_{i}^{*}\geq 0.$$

A new species may invade the community to establish coexistence equilibria with existing species, and existing species may be driven to extinction by new species or by environmental degradation. In a given environment shared by *S* species, the condition for equilibrium coexistence $n_{i}^{*}$ > 0 is achieved in a trade‐off between intrinsic fitness and competitive impact. A high intrinsic fitness is measured in high *R*_*i*_, for an intrinsically “fast” lifetime productivity that contributes to high *k*_*i*_. A high competitive impact is measured in high α_*ji*_−α_*ij*_, for a “dominant” competitor. Fast‐subdominant species (high *R*_*i*_, α_*ji*_−α_*ij*_ < 0) may thereby coexist with slow‐dominant species (low *R*_*i*_, α_*ji*_−α_*ij*_ > 0). On the standard assumption that traits carry costs, species have limited potential to combine a high intrinsic productivity with a high dominance (high *R*_*i*_, α_*ji*_−α_*ij*_ > 0). Persistence is possible for some slow‐subdominant species (low *R*_*i*_, α_*ji*_−α_*ij*_ < 0), though necessarily at lower abundances than those of species that are faster or more dominant. The coexistence equilibrium for a slow‐subdominant species is straightforwardly predictable from Eq. (1) if it functions as a fugitive to the other two species, meaning that it has no competitive impact on them. Then a slow‐dominant species‐1 coexists with a fast‐subdominant species‐2 at dynamic equilibria given by Eq. (2), and a slow species‐3 having fugitive traits α_13_ = 0 and α_23_ = 0 coexists amongst them at its dynamic equilibrium given by:

(3) $$n_{3}^{*}=k_{3}-\alpha_{31}\cdot n_{1}^{*}-\alpha_{32}\cdot n_{2}^{*}\;\text{for}\;n_{3}^{*}\geq 0.$$

**Figure 1:**
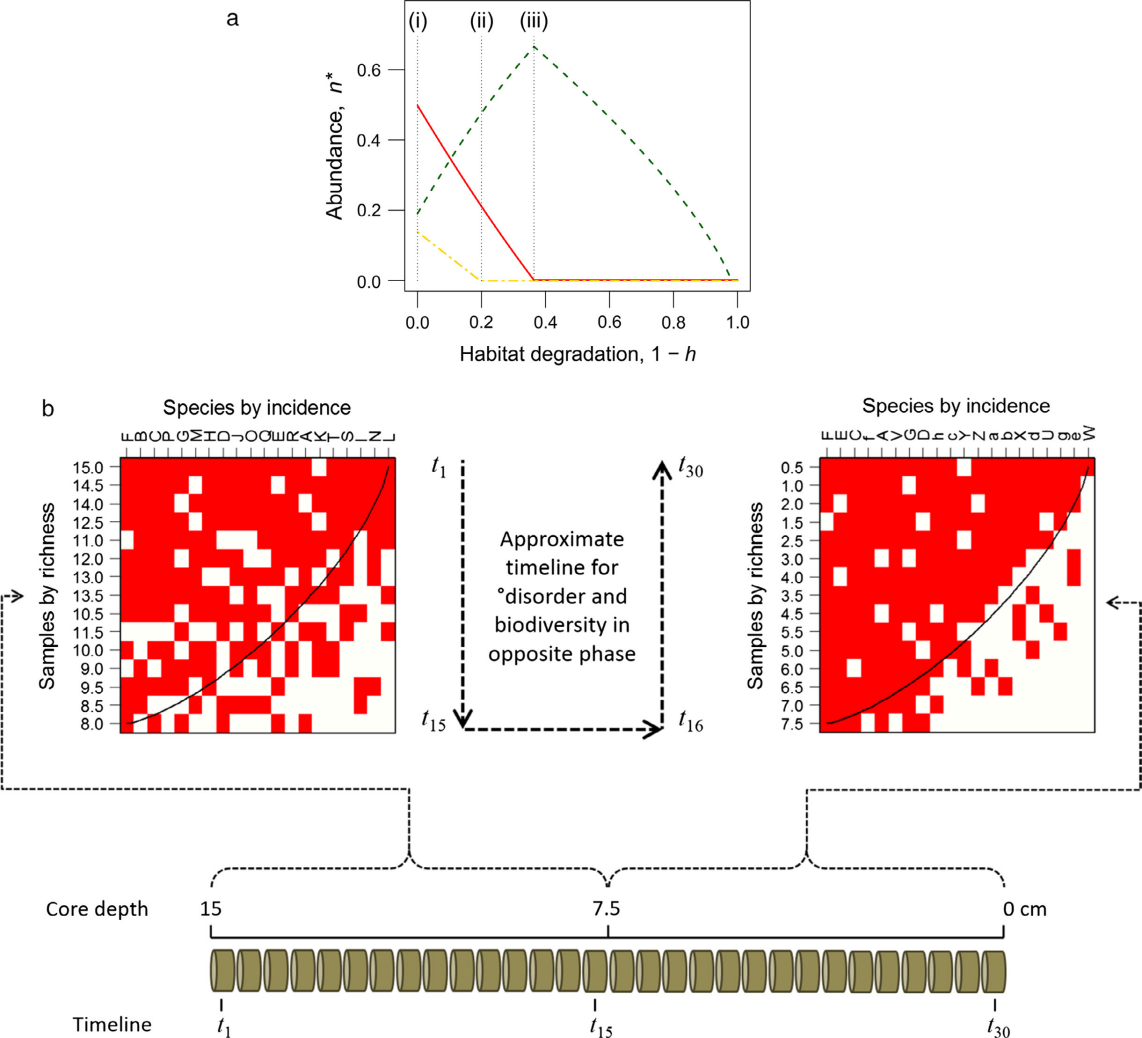
Dynamic concepts. (a) Phase plane of sequential extinctions of three species under increasing degradation of habitat *h*. Traces show Methods Eq. (1) at Eqs. (2) and (3) equilibria. (i) Initial prevalence of a keystone competitor (red continuous line); (ii) extinction first of the canary species (yellow dot‐dash); (iii) peak abundance of the weedy species (green dashed line) with extinction of the keystone. Keystone, weed and canary respectively take *c *= (0.20, 1.60, 0.18), *d *=* *0.1, α_1*i*_ = (1.00, 0.01, 0.00), α_2*i*_ = (1.50, 1.00, 0.00), α_3*i*_ = (0.50, 0.30, 1.00). (b) Schematic of two incidence matrices constructed from core samples and sorted by richness and incidence, demonstrating °disorder and biodiversity changing in opposite phase. Each matrix is 65% full (convex line indicates perfect species packing) with 20 letter‐coded species that are present (red) or absent (white) in 15 samples provided by the depth‐tagged core sections (brown). Biodiversity is calculated from the most recent core section in each matrix. In this example, the left matrix shows a high °disorder at 43.6°, and its most recent section (at 8 cm) is its least diverse with 6 species; the right matrix shows a low °disorder at 21.8°, and its most recent section (at 0.5 cm) is its most diverse with 19 species.

Habitat degradation has an ordered effect on these coexistence equilibria. As *h* reduces for all three species, vulnerability to extinction takes the order: slow‐fugitive > slow‐dominant > fast‐subdominant (Fig. 1a). Fast‐subdominants function as weedy species in being most resilient to exogenous forcing. Slow‐dominants function as keystone species by regulating the dynamics of all those beneath them in the dominance hierarchy, and their demise releases previously subdued species to proliferate in the community. Slow‐fugitives function as canary species in combining the least resilience with the least impact on community dynamics. The demise of the keystone may allow the weed to bloom in a smooth change of steady state (as Fig. 1a), or in a discontinuous switch characteristic of multiple stable states (if α_*ij*_ are themselves functions of *n*_*i*_).

Identification of weed, keystone, and canary species is problematic for diatom and chironomid communities. Although many taxa have known ecological preferences, most have community‐specific competitive abilities and reproductive rates presenting little scope for identification of indicator species. Even without knowing how individual species function, however, we find a community description closely allied to functional roles in the analysis of compositional disorder, which measures unpredictability in community composition.

### Quantification of compositional disorder

The ordering of species incidence in a community is characterized by nestedness (Patterson and Atmar 1986). For a strongly nested community, species composition is largely predictable from knowledge of the least frequently present species, since any samples from the community taken at different points in space or time tend to share all of the more frequently present species. Conversely, a weakly nested community has a less predictable composition, because any samples tend to share fewer species. Nestedness is usually measured across space (Bloch et al. 2007), or spatiotemporally (Heino et al. 2009, Florencio et al. 2011). It has also been applied to communities sampled repeatedly through time (Elmendorf and Harrison 2009, Angeler 2013), and here we apply it serially to simulated and empirical lake‐sediment cores sampled for their diatom or chironomid communities in replicate sections through a multi‐decadal timespan.

Nestedness is measured by units of “temperature,” on a continuous scale between 0° for a completely nested community and 100° for a completely unnested community (Rodríguez‐Gironés and Santamaría 2006). This calibration provides an analogy with °C of water in liquid phase, between phases of maximal structure as ice and maximal disorder as vapor. Accordingly, nestedness temperature rises with compositional disorder, from none at 0° with less species‐rich samples containing only higher‐incident species, to complete at 100° with no relation of species richness to incidence. We therefore refer to nestedness temperature henceforth as “°disorder.”

Calculation of each °disorder value requires an incidence matrix of *m* samples each containing one or more of *n* species. We used *m *=* *15 sequential core sections at 1–2 year intervals to construct each 15 × *n* matrix of 0/1 values recording absence/presence of each species in each sample. Matrix elements are shuffled to sort rows by richness, with more species‐rich samples towards the top, and columns by incidence, with more frequently present species towards the left. We used R function “nestedtemp” in library(vegan) to maximize species presences towards the top left and absences towards the bottom right (Oksanen and Carvalho 2013, R Core Team 2013). The threshold of perfect fill dividing these extremes is defined by the “fill line”: *y *= (1−[1−*x*]^*p*^)^1/*p*^ for matrix coordinates (*x*,* y*), with *p* selected to create a curve that covers the same area as the proportion of presences. Any presence to the right of the fill line, or absence to the left, is then a “surprise” observation relative to a cold matrix, and °disorder is calculated as a function of the sum across all surprises of squared deviations from the fill line. A low °disorder is obtained when presences pack towards the left and top such that the species composition of each sample is largely nested in the sample above it. A higher °disorder results from a less nested turnover of species between top and bottom samples.

### Relationship of °disorder with biodiversity

We measure biodiversity in both species richness, which disproportionately favors rare species, and Hill's (1973) diversity index *N*_2_ (henceforth referred to as “biodiversity”), which disproportionately favors common species (Jost 2006). Hill's *N*_2_ is commonly applied to fossil communities (e.g., Wang et al. 2012). Its value increases with species richness and evenness in species abundances: $N_{2}=1/\;\sum_{i=1}^{S}p_{i}^{2}$, where *p*_*i*_ is the proportional abundance of species *i* in a community of *S* species, with abundance given by the square‐root of percentage presence. In analyses of sediment cores, each value of °disorder was paired with a measure of biodiversity or species richness, taking its value from the most recent core section of the set of 15 consecutive sections used to calculate °disorder. It is therefore the biodiversity resulting from the °disorder.

The correlation of °disorder with biodiversity (or species richness) informs on the relative incidences of frequently present species and ephemeral species. Fluctuations across years that occur in opposite phase indicate a diminution of biodiversity during high °disorder and accumulation during low °disorder, which favors the frequently present species. This is demonstrated by the two matrices in Fig. 1b. Each matrix has had its elements shuffled to sort samples (rows) by richness and species (columns) by incidence, which in this illustration of opposite phase coincides with an approximately anticlockwise timeline. The left matrix shows a high °disorder = 43.6°, with the species turnover resulting in a diminished diversity of 6 species in its most recent section. Those species remain to enter the right matrix, becoming the core of an ordered accumulation that produces a low °disorder = 21.8° and a raised diversity of 19 species in its most recent section. Replicates of like pairs {43.6, 6}, {21.8, 19} would yield a negative correlation of °disorder with biodiversity, as frequently present species accumulate between peaks of species richness at the expense of turnover in more ephemeral species. In contrast, ephemeral species accumulate when °disorder and biodiversity fluctuate in same phase. A reversal of the Fig. 1b timeline demonstrates same phase, with an ordered loss of frequently present species in the right matrix being followed by disordered gain of ephemeral species in the left matrix. Replicates of like pairs {21.8, 6}, {43.6, 19} then yield a positive correlation of °disorder with biodiversity. Alternatively, a disordered timeline with negligible correlation would mean no consistent pattern of °disorder with biodiversity loss or gain.

### Interpretation of functional categories from °disorder‐biodiversity correlations

We built the conceptual model of community dynamics into an agent‐based simulation of a community that was designed to comprise populations of canary, keystone, and weedy species with Eq. (1) dynamics. Its purpose was to map these functional categories onto the frequently present species and the ephemeral species, whose relative incidences are detected by correlations of °disorder with biodiversity as described in the previous section. A good mapping of negative correlation during keystone prevalence (turning positive during weed prevalence) gives us reason to interpret a switch to negative correlation in empirical time series (where we have limited knowledge of functional categories) as a diagnostic signal of impending loss of keystones and critical transition to weeds. To achieve the mapping, the simulation of exogenous forcing needed only to force changes in the relative abundances of the different functional categories, not to create a critical transition per se. In a first period, we toggled the community‐wide death rate (*d* in Eq. (1) above) in order to induce the short‐term fluctuations in biodiversity and °disorder characteristic of empirical data. In a second period, we raised mean *d* in order to intensify the forcing. These stresses on the community induced a sequence of steady‐state responses in functional composition, from which we could test for community‐level signals in °disorder‐biodiversity correlations. The simulation developed on Doncaster (2009) as detailed in Appendix S1, with the code in Matlab 7.8.0 given in Metadata S1 and Data S1.

### Empirical datasets for testing predictions

Predictions from the simulation model were tested against empirical datasets on diatoms and chironomids from lake Erhai (Yunnan Province) and lake Taibai (Hubei Province) in China, which both underwent transitions. We have insufficient information on drivers or evidence of hysteresis to identify the type of transition sensu Capon et al. (2015). Instead, we construct our analysis on two reasonable assumptions: (1) The data show transitions of some kind, which were critical in taking the ecosystem to a new and sustained state of changed biodiversity, evidenced in statistically significant break points (sequential *t*‐tests plus arima forecasting) and corroborated by other studies; (2) structural changes within the system will precede any such critical transition in biodiversity (a generally accepted view). The transitions and structural changes in diatom and chironomid communities of Erhai and Taibai were compared to those of lake Longgan (Hubei‐Anhui provinces) with no critical transition.

Separate sediment cores were obtained for diatoms and chironomids from the deepest profundal zone of each of lakes Erhai (~21 m depth), Taibai (~3 m depth), and Longgan (~3 m depth). For each core, consecutive sections at intervals of 0.5 cm (Erhai and Taibai) or 1 cm (Longgan) throughout its length were dated by radionuclides (^137^Cs and ^210^Pb), from late 19th or early 20th century to early 21st century. Wang et al. (2012) describes preparation and preservation of diatoms, and Brooks et al. (2007) describes preparation and preservation of chironomids.

At each time‐tagged depth point up to the most recent date, biodiversity was calculated for that depth point, and °disorder was calculated for that depth point combined with the 14 prior depth points available below it. We used two methods for quantitative analysis of discontinuities in the time series that could be attributed to critical transitions. Dates of significant break‐points were identified by sequential *t*‐statistics on first‐order autoregressive models of time‐series means (NOAA software set for a significance threshold *α *= 0.05 sustained through at least 5 depth points with Huber's weight parameter of 1: Rodionov 2004, 2006). Forecasting models of °disorder and biodiversity were identified by autoregressive integrated moving average with R function arima(*p*,* d*,* q*), in which *p* is the autoregressive order, *d* the degree of differencing, and *q* the moving‐average order.

We prospected for a diagnostic signal of change in community structure that might precede the critical transition, by sequentially correlating °disorder with biodiversity. Pearson's product‐moment coefficient was calculated on consecutive °disorder‐biodiversity pairs through the time series using first differences between values. For example, Erhai diatoms had dated values of °disorder and biodiversity: {41.35, 18.64}_1898_, {38.59, 21.71}_1900_, {36.98, 19.90}_1903_ …, with corresponding first differences: {−2.76, 3.07}_1898_, {−1.61, −1.81}_1900_, …. For each dated core section, °disorder was correlated with biodiversity on two datasets: (1) the set of 15 points comprising that section and the 14 sections immediately preceding it, to give a coefficient for a putative “pretransition” period; (2) the complementary set of all earlier sections than these, to give a coefficient for a putative “lead‐in” period. A sensitivity analysis indicated that 15‐row incidence matrices maximize the magnitude of correlation for the putative pretransition period (Appendix S2: Fig. S1).

Neither the nonindependence of sequential observations, nor the compaction of the sediment core over time (Appendix S2: Fig. S2), compromise the correlations, which require independence only amongst residuals (Appendix S2: Fig. S3). Early‐warning signals of state change were identified from differences in the sign and magnitude of the correlation coefficient measured in discrete segments of time series, not from year‐on‐year evolution of the coefficient. For dated sections of the Erhai core it was nevertheless possible to consolidate the more recent and less compacted samples in order to create a reduced dataset of diatom abundances at 30 time points approximately equally spaced by 3–5 yr. This dataset yielded 16 consecutive time points from 1944 to 2005 with °disorder and biodiversity values, for comparison with the complete dataset.

## Results

### Diagnostic signal of keystone dominance in the simulation

**Figure 2:**
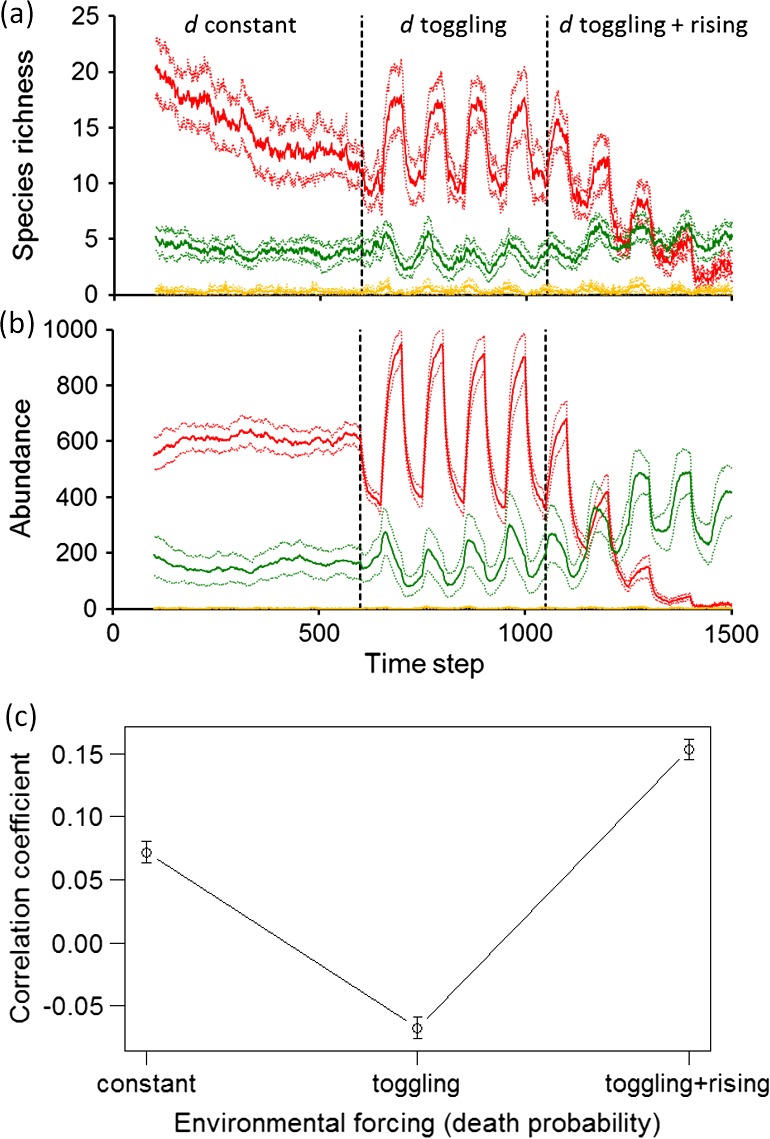
Simulated community exposed to consecutive periods of environmental forcing. An initial period of agents having constant death probability *d* is followed by a period of toggling *d* around the same mean as for the initial period, followed by a period of toggling *d* around a rising mean. All responses are means ± 95% c.i., *n *=* *15 replicate communities. (a) Number of species, and (b) numbers of individuals, of keystones (red, initially most numerous), weeds (green) and canaries (yellow, always rarest); (c) Product‐moment correlation of °disorder with Hill's diversity index *N*_2_.

Dynamics were simulated over time for a functionally structured community responding to three stages of environmental forcing modeled by death probability *d*. Keystone species were sustained through a middle period of *d* toggling between high and low values (Fig. 2a and b). Keystones increase slightly in numbers of species and abundance at the expense of other functional categories, compared to the initial period of constant *d*. The final period of rising *d* favors a marked increase in species richness and abundance of weeds at the expense of keystones. Canary species constantly enter the open system and go rapidly extinct without gaining predominance.

In response to these compositional changes, the correlation of °disorder with biodiversity switches from positive during the constant‐*d* period to negative during the toggling‐*d* period (Fig. 2c). The negative coefficient results directly from toggling, not from the change to toggling from constant‐*d* (Appendix S1). High death probability induces low biodiversity and high °disorder, and vice versa for low death probability, as conceptualized in the Fig. 1b timeline. The final period of *d* toggling and rising causes a switch back to strongly positive correlation, which is consistent with reversal of the Fig. 1b timeline.

These outcomes identify the frequently‐present species that do well during a period of negative correlation as the keystone species, and the ephemeral species that do well during positive correlation as the weedy species. Insofar as the negative correlation signals a prevalence of keystone over canary species (which never do well), it provides early warning of the critical transition to come when keystone dominance fails and the community collapses to residual weedy species.

### Critical transitions in paleo time series

**Figure 3:**
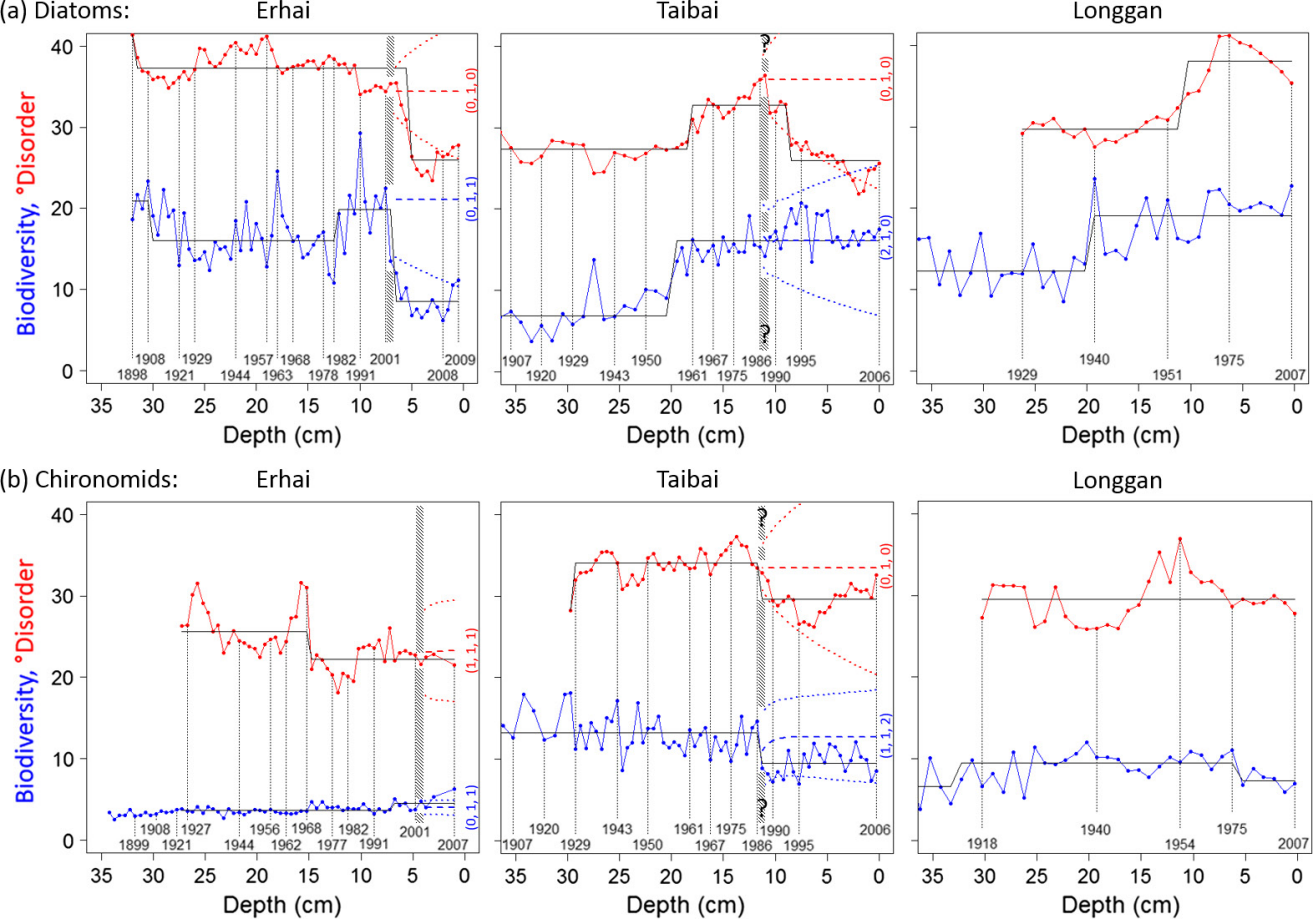
Temporal changes in °disorder (red) and biodiversity (blue), at Erhai, Taibai, and Longgan lakes. (a) Diatoms; (b) chironomids. Each core was sampled at 0.5‐cm depth intervals. Black stepped lines show means and significant break‐points identified by sequential *t*‐tests (*P *<* *0.05). Colored dashed and dotted lines show the arima(*p*,* d*,* q*) forecast and its 95% prediction intervals, after 2001 for Erhai and after 1986 for Taibai (vertical hashed bands, equivocal transitions for lake Taibai).

Evidence for critical transitions in diatoms and in chironomids was sought from information on community composition as well as analyses of break points and forecasts. The cores showed °disorder and biodiversity fluctuating and undulating through relatively constant long‐term trends, in lake Erhai up to c. 2001 and in lake Taibai up to c. 1986 (Fig. 3). Prior to 2001, Erhai had shown relatively little overall change in biodiversity or °disorder for diatoms or chironomids. Prior to 1986, Taibai had shown gradually rising biodiversity and °disorder of diatoms, but little overall change in chironomids. Longgan showed gradually rising biodiversity and °disorder of diatoms from the 1940s to 1970s, but little overall change in chironomids.

#### Erhai diatoms

The total of 156 species identified from 24 genera in 126 cross‐section samples were dominated by planktonic forms. Amongst these, *Fragilaria crontonensis* is a eutrophication indicator (Beeton 1965) which began increasing linearly from about 1960, while other typically eutrophic species including *Cyclostephanos dubius* and *Aulacoseira ambigua* increased linearly over the length of the core. A dramatic loss of biodiversity in 2001 in a critical transition to sustained alternate state, after at least a decade of rising variance and skewness in biodiversity, coincided with increases in *F. crontonensis* and *A. ambigua* (detailed in Wang et al. 2012). Over much of the 8 yr recorded at Erhai since 2001, diatoms remained below their 95% prediction limits for both °disorder and biodiversity, with suppressed variance in biodiversity (Fig. 3).

#### Taibai diatoms

Forty species from 19 genera in 74 cross‐sections comprised mainly facultative planktonic species (e.g., *Aulacoseira granulate*) and periphytic species (e.g., *Gyrosigma acuminatum*) until the 1950s when the lake started to become eutrophic. From the 1950s to 1970s the lake diminished to less than half its original size and phosphorus concentrations rose markedly, associated with agricultural land reclamation and introduction of commercial fishing stocks (detailed in Yang et al. 2008). Several dams were built in the upper reach of the catchment during the 1950s, precipitating marked changes in the hydrological conditions of the lake. From the mid‐1980s the lake began to receive wastewater effluent from chemical factories (detailed in Zhang et al. 2012). Macrophytes were abundant in the lake prior to the 1980s but subsequently became localized and have been absent from 2006 in the now hypereutrophic conditions dominated by algae (Zhang et al. 2012). The core provides equivocal evidence of a critical transition for diatoms in 1986. Over the 20 recorded years at Taibai since 1986, diatoms repeatedly fell below their 95% prediction limits for °disorder while biodiversity fluctuated around a relatively constant long‐term average (Fig. 3).

#### Longgan diatoms

A total of 68 species from 23 genera were identified in 41 cross‐sections. They comprised mainly facultative planktonic, benthic, and epithetic taxa (e.g., *Cocconeis placentula*), with very few planktonic species. Longgan is a shallow mesotrophic lake some 12 times larger than the hypereutrophic Taibai lake, the upper part of which drains into it. As with Taibai, its extent has been reduced from an originally larger size by land reclamation since the 1950s. After the 1970s, despite a rise in total phosphorous, epiphytic diatoms increased conspicuously indicating development of macrophytes. Total phosphorous is now 40% of its concentration in late Taibai, and submerged aquatic plants are abundant though showing signs of a gradual decline (Yang et al. 2008, Zhang et al. 2012).

#### Erhai chironomids

A total of 19 species from 16 genera were identified in 64 cross‐sections. These species feed on detritus and benthic algae including benthic diatoms. Numbers of chironomid head capsules had declined linearly from the 1960s onwards, suggesting a build‐up of hypolimetic anoxia. By 2001, previously rare taxa such as *Chironomus plumosus*‐type and *Tanytarsus mendax*‐type began to increase in abundance, exploiting niches created by eutrophication. The number of head capsules continued to decline as eutrophication continued up to and beyond the most recent core date of 2007. Conversely, biodiversity increased above the 95% prediction interval, albeit with very low numbers of head capsules overall, likely due to the lack of a profundal community allowing dominance of the more diverse littoral habitats (Fig. 3).

#### Taibai chironomids

A total of 50 species from 33 genera were identified in 74 cross‐sections. The chironomid stratigraphy had undergone a significant change in composition after 1950, reflecting a major deterioration in water quality. Taxa that dominated pre‐1950, such as *Paratanytarsus*,* P. penicillatus*‐type, *Polypedilum nubeculosum*‐type, and *Dicrotendipes nervosus*‐type are indicative of relatively clear water conditions with a reasonable macrophyte density and/or species richness (Langdon et al. 2010). Post 1950, these taxa diminished in abundance and some were almost lost from the record. They were replaced by an increasing abundance of taxa with a higher nutrient tolerance, including *Chironomus plumosus*‐type, a taxon commonly used as an indicator for eutrophic/anoxic conditions (Brooks et al. 2007), and *Microchironomus tabarui*‐type, *Procladius*,* Propsilocerus akamusi*‐type and *Tanypus*. The core again provides equivocal evidence of a critical transition for chironomids in 1986, with a noticeable drop in both °disorder and biodiversity which however remains largely within 95% prediction intervals (Fig. 3).

#### Longgan chironomids

A total of 35 species from 24 genera were identified in 45 cross‐sections. Chironomid composition from the 1950s onwards was dominated by *Paratanytarsus penicillatus‐*type and *Cricotopus sylvestris‐*type taxa associated with the clear‐water state characteristic of macrophyte development since the 1970s (detailed in Zhang et al. 2012).

These lake‐ and taxon‐specific changes in composition in relation to break‐points and deviations from forecasts provide evidence of critical transitions to sustained alternate states in Erhai in 2001 for both diatoms and chironomids, and equivocally in Taibai in 1986 for diatoms and chironomids. A detrended correspondence analysis provides corroboratory evidence (Appendix S3). For the Erhai communities of diatoms and chironomids, the ordination of DCA shows clearly separate groupings after ~2001; Taibai diatoms show a separation after ~1990, and again after ~2000, while Taibai chironomids show no separation (Fig. S1). These interpretations are consistent with those of published analyses of biodiversity metrics in the lakes (Yang et al. 2008, Wang et al. 2012, Zhang et al. 2012). We henceforth refer to the periods 2001–2009 at Erhai, and 1986–2006 at Taibai, as the “transitioned” periods. Although the time series for lake Longgan have wider sampling intervals, and therefore contain less information, this mesotrophic and macrophyte‐dominated lake provides the best available comparator with no evidence of eutrophication or a critical transition in the 20th and 21st centuries (Fig. 3 and Appendix S3: Fig. S1; consistent with Yang et al. 2002, 2006).

### Diagnostic signal of approaching critical transitions in paleo time series

**Figure 4:**
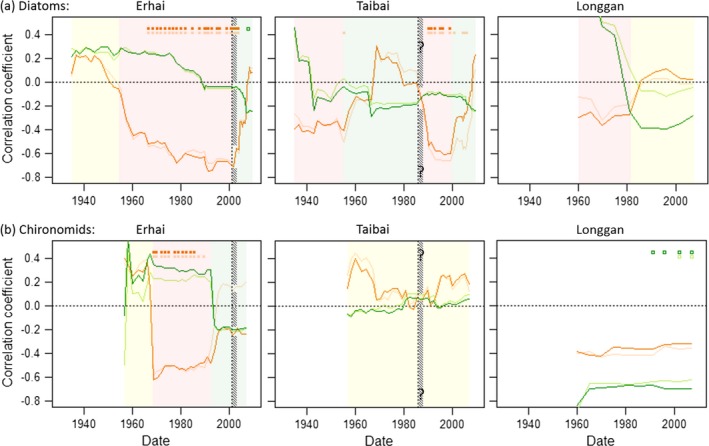
Sequential product‐moment correlations on first differences of °disorder with biodiversity (dark traces) and with species richness (pale traces), at Erhai, Taibai, and Longgan lakes. (a) Diatoms; (b) chironomids. Green traces show the putative lead‐in period until 15 core sections before the *x*‐axis date, and orange traces show the putative pretransition period from that point to the *x*‐axis date. In each graph, top rows of dots identify dates with *P *<* *0.05; horizontal dotted black line indicates coefficient of zero, and vertical hashed band indicates date of 2001 and 1986 critical transition for Erhai and Taibai respectively. Colored backgrounds indicate approximate time partitions in which environmental conditions favored canary (yellow), keystone (red) and weed (green) species, as suggested by the sign and magnitude of the putative pretransition coefficient relative to the lead‐in coefficient.

Where regime shifts occurred, the pretransition period had a relationship of biodiversity to °disorder that was characterized by strongly negative correlations, following neutral or positive correlations for earlier lead‐in periods (Fig. 4). The following analyses report correlations on first differences of °disorder and biodiversity, in accordance with the forecast models in Fig. 3 indicating stationary time series only in the means of first differences (Appendix S2: Fig. S2). Plots of the evolution of pretransition and lead‐in coefficients over time (Fig. 4) show strong diagnostic signals for Erhai diatoms and chironomids from three decades prior to 2001, and for Taibai diatoms from one decade prior to 2000. Consider for example the diatoms in lake Erhai (Fig. 4a left‐hand graph). Had a core been taken here in 1968, it would have already detected a significant switch to strongly negative correlation of −0.51 for the 15 sections 1937–1968 (orange pretransition trace at 1968) from a weakly positive correlation of 0.24 for the preceding 31 sections ~1850–1937 (green lead‐in trace at 1968). The putative pretransition period had had an increasingly negative correlation coefficient up to 1968, which would have continued to strengthen had subsequent cores been taken over the next three decades up to the 2001 critical transition, after which it would turn weakly positive.

#### Erhai diatoms

Correlations of °disorder with biodiversity indicate changes in the community consistent with a predominance of weed species under eutrophication from 2001, following a collapse of previously dominating keystone species. From 1966 until 2001, a significantly negative correlation for the putative pretransition period steadily strengthens in magnitude and in difference to the weakly positive correlation for the putative lead‐in period (Fig. 4a, left‐hand graph). Multiple regression analyses of 1898–1966 “lead‐in,” 1966–2001 “pretransition” and 2001–2009 “transitioned” time partitions found that only the pretransition negative correlation had a significant coefficient (Appendix S2: Figs. S3 and S4). These patterns depend very little on species abundances, given the similarity between biodiversity and species richness. The dataset at equally spaced time points showed a similar relationship to that of the full dataset. Its most parsimonious time‐series model was arima(0, 1, 0) for both °disorder and biodiversity, indicating stationary time series in the mean of first differences. For a lead‐in time partition up to 1963, multiple linear regression on first differences showed no significant effect of °disorder on biodiversity (*t*_4_ = 0.198, *P *>* *0.8, model *r* ^2^‐adjusted <0.001) after removal of non‐significant date main effect and interaction. For the pretransition time partition 1963–1998, multiple linear regression showed a negative main effect of °disorder on biodiversity (*t*_8_ = −2.340, *P *=* *0.047, model *r* ^2^‐adjusted = 0.332) after removal of nonsignificant date main effect and interaction (Appendix S2: Fig. S5).

#### Taibai diatoms

Correlations of °disorder with biodiversity indicate community‐level changes consistent with eutrophication from the 1950s to 1990s and hypereutrophication from the 2000s. In the 1930s‐1950s, the relatively more negative pretransition than lead‐in coefficients prefigure the approaching eutrophication from the 1950s onwards (Fig. 4a, middle graphs). The subsequent change to a period of correlation coefficients with more positive pretransition than lead‐in values during the 1970s‐1980s happens at a time of growth in both °disorder and biodiversity (Fig. 3a, middle graph). It suggests a rising predominance of ephemerals following a demise of keystone species, as new weed and canary species contested for space in the diminished expanse of the now eutrophic lake. From 1986, a sharp drop in the pretransition correlation to significantly negative values reflects a sharp drop in °disorder against a relatively constant biodiversity. It suggests an accumulation of frequently present (keystone) species at the expense of ephemeral species in response to the appearance of chemical wastewater from the mid‐1980s. The decline in pretransition coefficients during the 1990s offers a strong early warning signal for the deterioration into hypereutrophic conditions that took place ~2000, which is not otherwise apparent from the time series of biodiversity. The hypereutrophication is reflected in the DCA ordination of Taibai diatoms, showing separate clusters either side of 2000 (Fig. S9). The most recent stage of rising pretransition correlation from ~2000 is consistent with the replacement of keystone by weed as algae dominated the system.

#### Erhai chironomids

From 1968 to the mid‐1990s, a strongly negative pretransition period differs clearly from a weakly positive lead‐in (Fig. 4b, left‐hand graph). As for the diatoms in lake Erhai, it indicates a community favoring accumulation of keystone species. The relationship likewise does not depend on knowledge of species abundances, as species richness gives negligibly different correlation coefficients. The coefficients cease to differ in the ~6 yr preceding the 2001 critical transition, suggesting a collapse of keystone species occurring at a mesotrophic state of the lake prior to eutrophication.

#### Taibai chironomids

At no time from the 1960s to the 2000s do the correlations of °disorder with biodiversity become strongly negative (Fig. 4b, middle graph). The apparent drop in chironomid biodiversity from 1986 (Fig. 3b, middle graph) has no detectable consequence for the correlation of °disorder with biodiversity. The more positive coefficients for putative pretransition than lead‐in periods around the 1960s and the 2000s are consistent with environments that favor loosely interacting species.

#### Longgan diatoms and chironomids

The °disorder‐biodiversity correlations indicate a shift away from favoring keystone species, strongly for diatoms from the 1980s, and weakly for chironomids from the earliest records in the 1960s, consistent with an increasing predominance of macrophytes (Fig. 4 right‐hand graphs).

## Discussion

We have identified a diagnostic signal of compositional changes in response to exogenous forcing of a simulated community, and observed it in diatom and chironomid communities preceding critical transitions. Further research needs to confirm that system transition dynamics cause the negative correlations of °disorder with biodiversity in the empirical data. Although we make a causal link by modeling the replacement sequence of canaries > keystones > weeds, in real communities anthropogenic forcing may influence the correlations independently of transition dynamics. For example, communities with less marked competitive asymmetries than those modeled here may reduce instability in a stressed community by supplanting ephemerals with frequently present species other than keystones. In communities of species that compete for similar resources, however, biodiversity depends strongly on the asymmetries characteristic of competition‐growth tradeoffs. For communities of aquatic organisms competing for similar nutrients in well‐mixed lakes, this model becomes increasingly appropriate as environmental stressors further reduce the spectrum of available resources. The expression of the diagnostic signal may also depend on the type of transition (e.g., fold, gradual or no bifurcation: Boettiger et al. 2013). Future models could explore this possibility by directly linking critical transitions to °disorder‐biodiversity correlations. The lake datasets show signals prior to both eutrophication and hypereutrophication, which may involve different types of transition. Further empirical analyses in progress on time series of oceanic phytoplankton suggest a potential wider applicability to different communities and dynamics from those modeled and tested here. There is a general need for more time series with sufficiently long sequences of unchanging dynamics to evaluate the likelihood of false positives. Our analysis of lake Longgan, for example, has smaller sample sizes than other lakes for calculating sequential correlations during the putative lead‐in period. The weakening magnitude of its correlations from putative lead‐in to pretransition nevertheless suggests opposing dynamics to those of lakes Erhai and Taibai during their approaches to known changes of state for both diatoms and chironomids (Fig. 4).

Metrics‐based early‐warning signals of flickering and critical slowing down in time series are sensitive to any increase in compaction of the time series with depth or changes in sedimentation rates over time (Carstensen et al. 2013). In contrast, tests for differences in the sign and strength of time‐paired correlations between discrete periods control for these temporal effects. The correlations of °disorder with biodiversity test fluctuations only for synchrony (none for the lead‐in, strong for the pretransition and transitioned periods) and phase (opposite phase for the pretransition, same phase for the transitioned period). Moreover, the use of first differences removes the potential for effects that would necessarily show in °disorder values themselves, for example in annual sampling from a multi‐annual cycle. The three decades of detectable pretransition at Erhai are characterized by both the biodiversity of diatoms and of chironomids fluctuating around stationary means in first differences. The negative correlation of °disorder with biodiversity for each taxonomic group is explicable as a repeated switching between species loss by turnover and gain by accumulation. The dynamic is consistent with a steady reduction in niche diversity, as canary species are flushed out by an increasing prevalence of slow‐dominant and fast‐fugitive species. This in turn sets up the conditions for community collapse, as on‐going habitat degradation forces out slow‐dominant species to release the fast‐fugitive “weedy” species.

The critical transition in lake Erhai was linked to rapid eutrophication driven by nutrient loading from agriculture combined with sewage effluent, water‐level changes and climate change (Wang et al. 2012). Reduced oxygen in hypolimnetic anoxia induced positive feedback in phosphorus, algae, and oxygen, followed by hysteresis. The negative correlation of °disorder with biodiversity during the approach to the critical transition became apparent in diatom and chironomid time series some 10–20 yr prior to the initiation of “flickering” identified in the same community of diatoms by Wang et al. (2012). Lake Taibai showed similar patterns in diatoms, preceding both eutrophic and hypereutrophic conditions. This makes a strong case for adding °disorder and its relationship with biodiversity to the armory of diagnostic tools for monitoring systems that might be prone to critical transitions. Although correlations alone cannot inform causality, they can alert ecosystem managers to potentially destabilizing dynamics in the repeating sequences of disordered losses of species and ordered gains during biodiversity fluctuations.

Amongst diatoms in the sampled lakes, *Aulacoseira* sp., *Cyclostephanos dubius*, and *Cyclotella* sp. could function as keystone species, being dominant in stable state and holding wide niches; nondominant species with narrow niches such as *Navicula* sp. *Nitzschia* sp. could function as canaries; and eutrophic species such as *Fragilaria crotonensis* could function as weeds. *Fragilaria* spp. tend to dominate in spring and *Cyclotella* spp. in autumn. Their segregation may reflect temporal partitioning in competition for resources, involving interference with access to fast‐renewing resources, or exploitation of slow‐renewing resources. The resultant decoupling of species weakens the correlation of °disorder with biodiversity. Thus the low magnitudes of correlations in the simulation reflect its probabilistic input settings for competition coefficients encompassing some pairs with low values for both α_*ji*_ and α_*ij*_. Although the temporal resolution of empirical core sections was too coarse to pick up seasonality, their high‐magnitude correlations are consistent with an annual scale of competition between species for slow‐renewing resources.

Amongst chironomids in the sampled lakes, taxa that typically dominate are *Procladius* and *Michrochironomus*, and hence they could be considered keystones; some Chironominae are typically ephemeral, such as *Einfeldia* and *Endochironomus*, which my function as canaries; *Chironomus plumosus*,* Polypedilum* and *Tanytarsus mendax* types have weedy attributes as they dominate in eutrophic conditions (Brooks et al. 2007). Chironomids have less seasonality than diatoms, and metrics‐based analyses of their time series have not yet been able to identify early‐warning signals of critical transitions.

Importantly, knowledge of these species‐specific functions is not necessary for the early detection of an approaching critical transition from species composition, because relevant responses to environmental stressors are picked up by the correlation of °disorder with biodiversity. The equally strong correlation of °disorder with species richness as with Hill's diversity index *N*_2_ suggests that detection of differences in correlation may only need data on species presence/absence and not abundances. With each value of °disorder requiring 15 time points in the analyses shown here, correlation of its temporal fluctuations with biodiversity requires a time series with at least twice as many observations, and longer still to detect changes in correlation. The biodiversity does not have to be large, however, as shown by the lake‐Erhai chironomids which averaged 5–6 species before the critical transition.

In conclusion, our study demonstrates a potential role of changing species composition in the approach to critical transitions, which is predictable on theoretical grounds and detectable with knowledge only of species incidence through time. By analyzing community composition over decadal to centennial time periods, we better understand the mechanisms driving a loss of resilience as communities shift outside safe operating spaces prior to a critical transition. To help ecosystem management, future research needs to develop mechanistic models that can simulate critical transitions as a function of the strength and constancy of environmental drivers.
