# Supplementary material for: Early warning of critical transitions in biodiversity from compositional disorder
Source: Ecology. 2016 Nov 3;97(11):3079–90. doi: 10.1002/ecy.1558 (PMC6849621; doi:10.1002/ecy.1558)
Supplement: Supplementary file 1 [file ECY-97-3079-s001.pdf]

# APPENDIX S1. Simulation of species composition under environmental stress

We developed an agent-based model of a community of functionally distinct organisms, in order to simulate changes in the relationship of °disorder to biodiversity as a function of environmental forcing. The model is designed to simulate competitive hierarchies within a trophic level, and not taxon-, location- or season-specific parameters. Here we describe the structure of the model, built in Matlab 7.8.0, and its parameterization with values listed in Table S1. Appendix S2 provides the coding for the simulation. Outputs are analyzed in the main text.

### *Model*

An open population occupied a size-limited environment of $K$ cells. Individuals in the population cycled through birth, self-replication and death, with competition within and between species for access to cells. All $N$ species in the pool of total available species were assigned the same intrinsic per capita death probability, $d$. Each of the $N$ species was assigned its own intrinsic per capita probability of self-replication, $c$, and $\alpha$ coefficients for probabilities of winning contests with every other species. The relationship between values of these two parameters allowed categorization of each species as a 'keystone' (strong competitive impacts on other species, low $c$), 'weed' (weak competitive impacts, high $c$), or 'canary' (weak competitive impacts, low $c$). The relative prevalence of these three types was mapped over three stages of environmental forcing. Environmental forcing was represented by an initial period of constant $d$, followed by a period of $d$ toggling between higher and lower values around the same mean as for the first period, followed by a final period of $d$ toggling with rising mean value. Coefficients of correlation between °disorder and biodiversity were analyzed for changes across these three periods.

### *Competition between species*

An $\alpha$ matrix of $N{\times}N$ correlation coefficients was created to define the probability of a species winning access to a cell in competition with any other species. For a given species $i$, column $\alpha_{.,i}$ contained all impacts imposed on others, and row $\alpha_{i,.}$ contained all impacts received from others. The diagonal element $\alpha_{i,i}$, representing intraspecific competition, was set to unity. Other values were populated by a stratified-random process that maximized the range of column and row means, $\alpha_{c,i}$ and $\alpha_{i,r}$ respectively. This was done by imposing a gradient in column means with a range $0 < h < 1$, and enforcing a minimum row means between 0 and $1 - h$. Each species $i$ had a net competitive ability $\bar{\alpha}(i) = (1 - [\alpha'_{i,r} - \alpha'_{c,i}])/2$, where $\alpha'_{c,i}$ and $\alpha'_{i,r}$ are column and row means rescaled to give the worst and best attacking species values of $\alpha'_{c,i} = 0$ and 1 respectively, and the worst and best defending species values of $\alpha'_{i,r} = 0$ and 1 respectively. Lowest and highest values of $\bar{\alpha}$ were obtained by species that were respectively weakest and strongest in both attack and defense.

### *Competition-growth trade-off*

A trade-off was forced on all species between competitive ability and capacity for self-replication, in order to reflect the reality of costly reproduction (Doncaster 2009) and to prevent any one dominant and quickly-replicating species from extirpating all others in the community. The trade-off was enforced for each species $i$ by assignment of its $c_i$ to a random value between a community-wide $c_{min}$ and a sigmoidal function of $\bar{\alpha}(i)$ defined by community-wide $c_{max}$ and $\beta$

defining the slope of the upper threshold of $c$ with $\bar{\alpha}$. The resulting matrix of $\bar{\alpha}$-$c$ values defined keystones (high $\bar{\alpha}$, low $c$), weeds (low $\bar{\alpha}$, high $c$), and canaries (low $\bar{\alpha}$, low $c$), as illustrated by the example plot in Fig. S1.

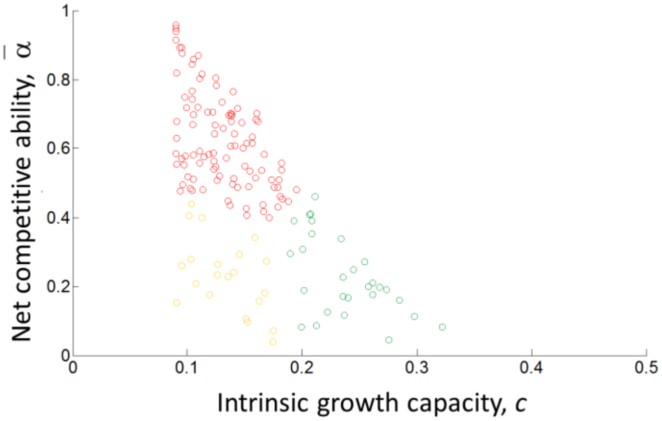


FIG. S1. Example plot from an $\bar{\alpha}$-$c$ matrix, identifying species as keystone (red), weed (green), canary (yellow).

### *Agent life cycle*

At each time step in the simulation each resident agent of species $i$ had probability $c_i$ of propagating a new agent, and then probability $d$ of death (which removed it from the cell). A newly propagated agent competed for access to one of the $K$ cells selected at random, and survived the time step only if the cell was empty or it succeeded in ousting a resident occupant. In either case it had first to beat other unattached agents, which depended on it having superior attacking ability. The probability of an unattached agent of species $i$ winning against an unattached competitor of species $j$ is $\alpha_{ji} /(\alpha_{ji} + \alpha_{ij})$. Its probability of then ousting a resident agent of species $j$ from an occupied cell is $1 - \alpha_{ij}$.

### *Species influx and outflux*

Each model run started with five agents of each of $n$ species chosen at random from the pool of $N$ species. Each time step allowed an influx of a further five agents of each of two randomly selected species. Species outflux in extinction was controlled by the community-wide $d$, which took values that reflected three stages in environmental forcing. It was held constant at $d_1$ during a first period from time step 0 to 599 (with data outputs recorded after the initial 150 time steps). This was followed by a second period from time step 600 to 1049 in which $d$ toggled above and below a constant mean = $d_1$ with amplitude $A$ and cycle length $T$. A final period from time step 1050 to 1500 sustained the toggling while mean $d$ rose at constant rate $\delta$ over time.

### *Analysis*

At each time step, biodiversity and °disorder (over the 15 preceding time steps) were calculated in accordance with the main-text Methods. At the end of a run, the program reported a triplet of coefficients for Pearson's correlation between °disorder and biodiversity for each of the three periods. A total of 100 replicates were run on each of 15 $\bar{\alpha}$-$c$ matrices.

TABLE S1. Parameter constants for the simulation run over three consecutive periods ($d$ constant, toggling, toggling plus rising).

| Parameter | Value | Meaning |
|---|---|---|
| $K$ | 1600 | Size of the environment in grid cells |
| *time* | 1500 | Time steps per run |
| $n$ | 20 | Initial number of species |
| $N$ | 150 | Total available species pool |
| $c_{min}$ | 0.1 | Minimum replication rate per capita per time step |
| $c_{max}$ | 0.5 | Maximum replication rate per capita per time step |
| $\beta$ | -18.0 | Slope of sigmoidal trade-off threshold between $\bar{\alpha}$ and $c$ |
| $d_1$ | 0.09 | Mean death rate per capita per time step during 1st and 2nd periods |
| $T$ | 100 | Cycle length of toggling death rate per capita |
| $A$ | 0.07 | Amplitude of toggling cycle |
| $\delta$ | 0.03 | Rate of increase in mean death rate per capita during 3rd period |

### *Sensitivity analysis*

Simulation outputs are reported for the parameter constants given in Table S1. This combination of values set an appropriate size of environment for sustaining a community of three types of species (keystone, weed and canary) through three stages of environmental forcing ($d$ constant, toggling, toggling plus rising). We also explored variations in $c_{min}$, $c_{max}$, and $d_1$ between zero and unity, and variations by orders of magnitude in values of $d_1$, $T$, $A$, and $\delta$. The patterns detected by analysis of variance (next section) were robust to these variations where they allowed the community to approach steady state, and where $T$ was sufficiently long to allow calculation of °disorder at high and low biodiversity. The magnitude of the correlation of °disorder with biodiversity was most influenced by $A$ during the period of $d$ toggling around a constant mean $d_1$ (more negative with higher $A$), and by $\delta$ during the final period of $d$ toggling around a rising mean $d$ (more positive with higher $\delta$).

### *Results*

Species of more keystone character tend to prevail in the period of toggling $d$ around constant mean $d_1$, and species of more weedy character tending to prevail in the final period of rising mean $d$ (main-text Fig. 2). A typical example of variation in agent abundances within and between species over the course of a run is shown in Fig. S2.

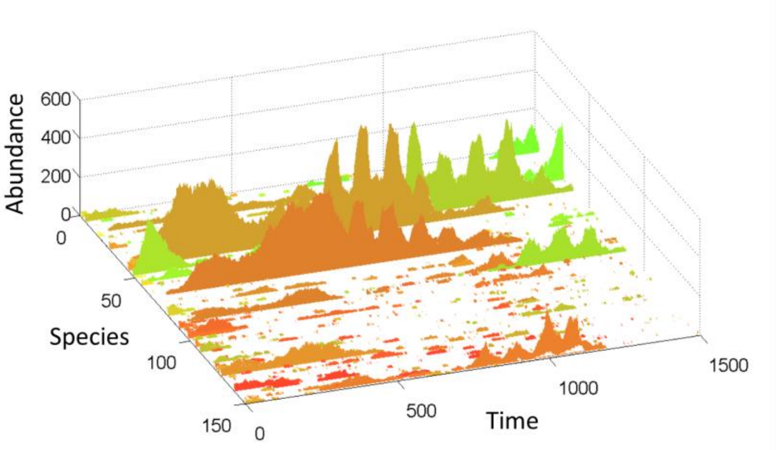


FIG. S2. Example of population structure evolving over one run. Abundances plotted for each of 150 species, in a color palette grading between red for keystone, green for weed, and yellow for canary. Time steps 0-599 have constant *d*; 600-1049 have *d* toggling around the same average; 1050-1500 have *d* toggling and rising.

Mean coefficients of the correlation of °disorder with biodiversity (main-text Fig. 2c, and Fig. S3) show a consistent pattern of initially positive coefficients during the period of constant *d* (overall mean coefficient 0.072), switching to negative during the period of toggling *d* (-0.067), and to more strongly positive during the period of toggling plus rising *d* (0.154).

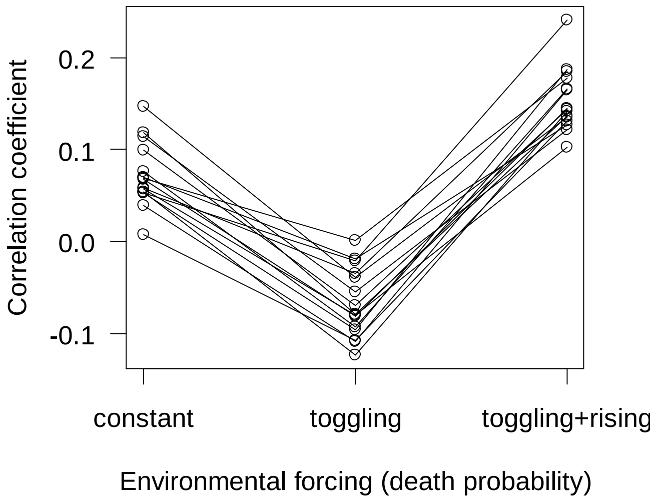


FIG. S3. Mean product-moment correlation coefficients of °disorder with biodiversity for 450 time steps in each of three periods of environmental forcing (as main-text Fig. 2c). Open circles show means for each of 15 replicate $\bar{\alpha}$-*c* matrices, with each triplet of coefficients based on 100 runs using Table S1 parameter values.

An influence of period on the correlation coefficient was tested with the total set of 1500 triplet coefficients in analysis of variance (function aov in R) using split-plot model:

$$\text{Coefficient} \sim \text{M*A} + \text{Error(M:A)}$$

with three levels of fixed period factor A crossed with 15 levels of random matrix factor M. This model showed a significant effect of period (Table S2, A effect: $F_{2,28} = 171.43$, $P < 0.0001$).

TABLE S2. Split-plot analysis of variance on the correlation-coefficient response, with period (factor A taking three levels: constant, toggling, and toggling+rising) tested on replicate $\bar{\alpha}$-$c$ matrices (factor M with 15 replicates), and each triplet of sequential coefficients based on 100 runs of the simulation using Table S1 parameter values.

| Term | d.f. | SS | MS | *F* | *P* |
|---|---|---|---|---|---|
| *Error*: M:A | | | | | |
| M | 14 | 2.22 | 0.159 | - | - |
| A | 2 | 37.37 | 18.686 | 171.43 | < 0.0001 |
| M:A | 28 | 3.06 | 0.109 | 4.04 | < 0.0001 |
| *Error*: Within | | | | | |
| Residuals | 4455 | 120.20 | 0.027 | | |

The period effect was subjected to *a priori* orthogonal contrasts (methods in Doncaster and Davey 2007). Period was partitioned into a contrast B between the 2nd period and pooled 1st with 3rd periods, and a contrast C (nested in B) between the 1st and 3rd periods, using the model:

Coefficient ~ M + B/C + Error(M:A)

The analysis showed a negative average coefficient for correlations of °disorder with biodiversity over the period of $d$ toggling around constant mean $d_1$, which differed significantly from the pooled positive averages for the periods of constant $d$ and rising mean $d$ (Table S3, contrast B, $F_{1,28} = 296.18$, $P < 0.0001$). Moreover, the period of rising mean $d$ had a significantly larger positive average than the period of constant $d$ (Table S3, contrast B:C, $F_{1,28} = 45.77$, $P < 0.0001$).

TABLE S3. For the Table-S2 period effect, analysis of orthogonal contrasts B (toggling vs. pooled constant and toggling+rising) and C (constant vs. toggling+rising).

| Term | d.f. | SS | MS | *F* | *P* |
|---|---|---|---|---|---|
| *Error*: M:A | | | | | |
| M | 14 | 2.22 | 0.159 | - | - |
| B | 1 | 32.37 | 32.370 | 296.18 | < 0.0001 |
| B:C | 1 | 5.00 | 5.002 | 45.77 | < 0.0001 |
| M:A | 28 | 3.06 | 0.109 | 4.04 | < 0.0001 |
| *Error*: Within | | | | | |
| Residuals | 4455 | 120.20 | 0.027 | | |

The negative correlation during the toggling period depended on the condition itself, not the change of condition. This was demonstrated by repeating the simulation with the period of constant $d$ replaced by toggling $d$ for all of the first 1049 time steps. Correlations had equally negative coefficients for time steps 600-1049 (sample of 4 matrices: $F_{1, 795} = 3.15$, $P > 0.05$).

### *Literature cited*

Doncaster, C. P. 2009. Ecological equivalence: a realistic assumption for niche theory as a testable alternative to neutral theory. PLoS ONE 4:e7460.

Doncaster, C. P., and A. J. H. Davey. 2007. Analysis of variance and covariance: how to choose and construct models for the life sciences. Cambridge University Press, Cambridge UK.
