# Supplementary material for: Early warning of critical transitions in biodiversity from compositional disorder
Source: Ecology. 2016 Nov 3;97(11):3079–90. doi: 10.1002/ecy.1558 (PMC6849621; doi:10.1002/ecy.1558)
Supplement: Supplementary file 2 [file ECY-97-3079-s002.pdf]

APPENDIX S2. Supplementary analyses of empirical time series

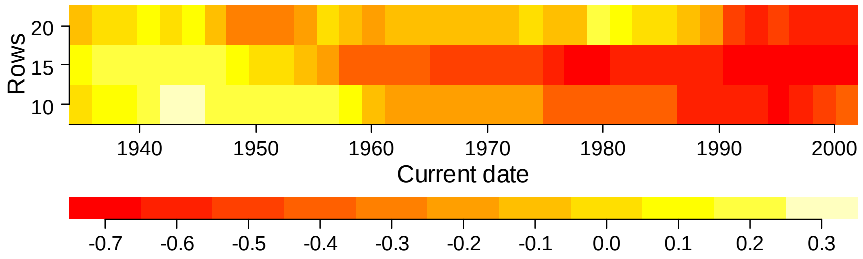

FIG. S1. Heat map for sensitivity of correlations to number of incidence-matrix rows, Erhai diatoms. Colors show coefficient magnitude for the correlation of biodiversity and °disorder on first differences, for the putative pre-transition period of 15 consecutive core sections up to the current date.

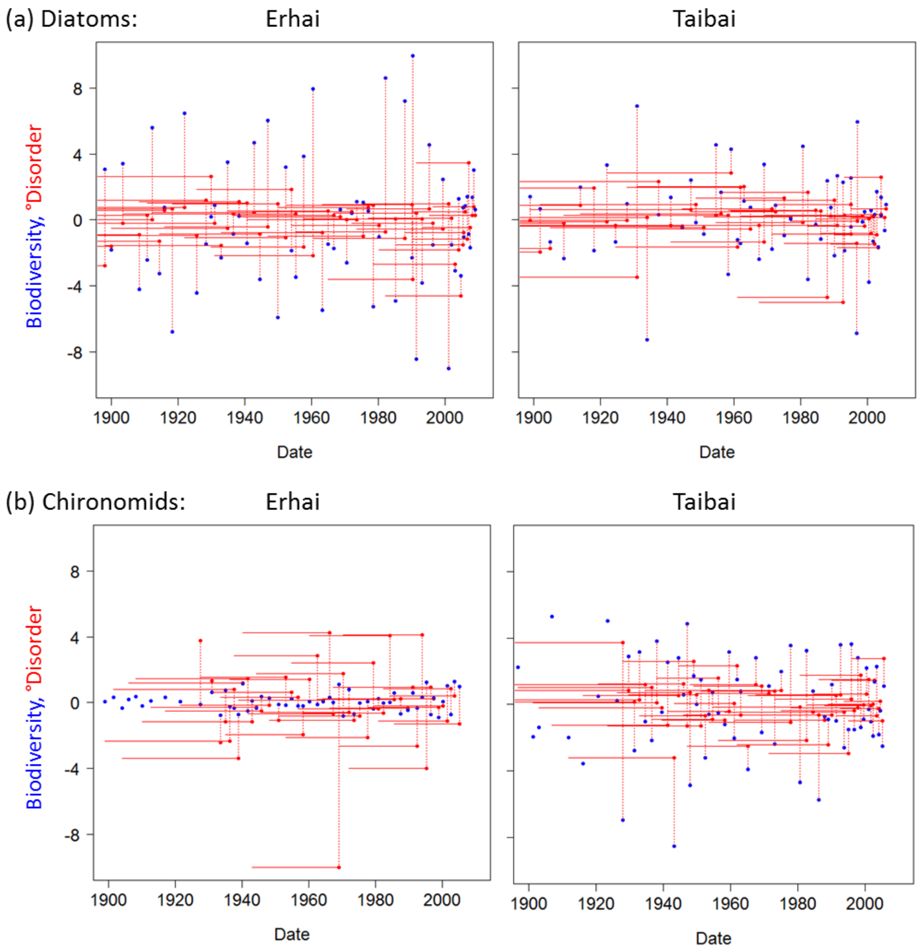

FIG. S2. First differences of °disorder (red) and biodiversity (blue) by date, at Erhai (left-hand graphs) and Taibai (right-hand graphs). (a) Diatoms; (b) chironomids. Each date-specific value of Hill's diversity index N_2 (blue dot) is a response to °disorder (red dot linked by red dotted vertical line). Each °disorder value is calculated over the preceding 15 core sections (red horizontal line covering 7.5 cm), which cover progressively shorter time segments with less compaction towards the present.

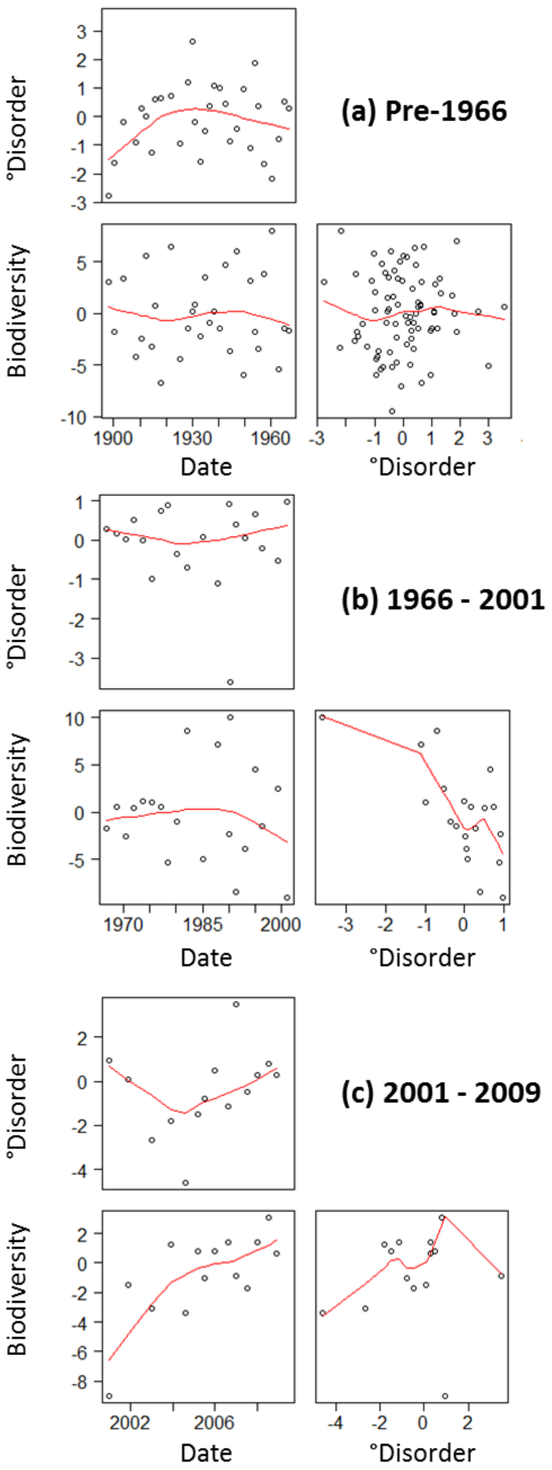

FIG. S3. Pairwise correlations of date, first differences in ºdisorder, and first differences in biodiversity, Erhai diatoms. The three time partitions are (a) lead-in, (b), pre-transition, and (c) transitioned, using the data from main-text Fig. 3a.

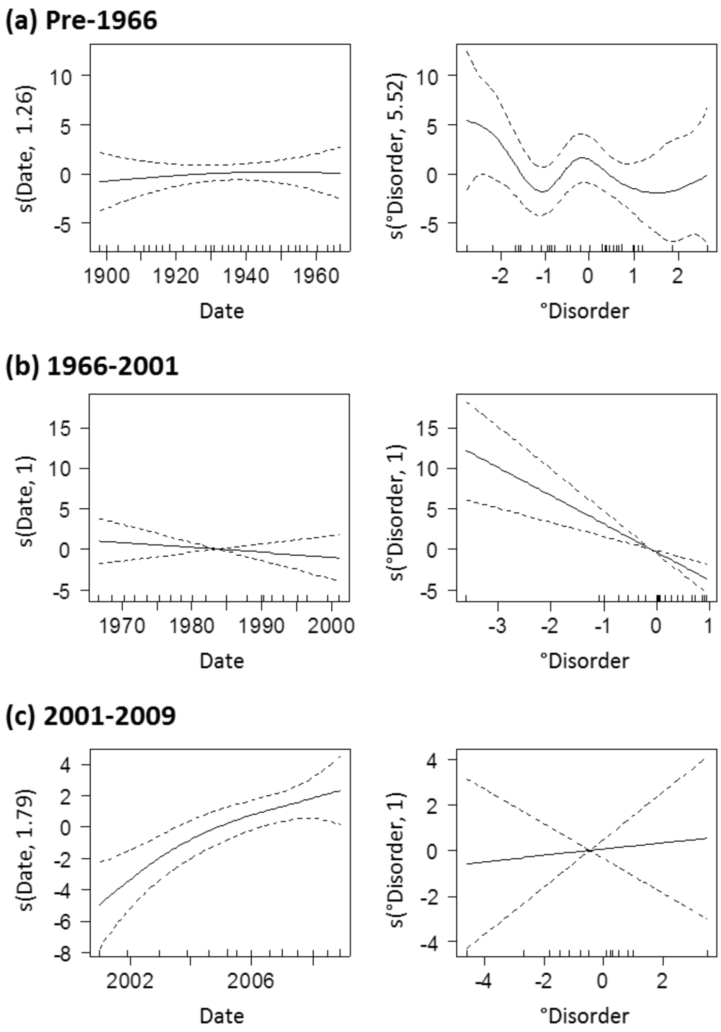

FIG. S4. Non-parametric smoothing in generalized additive models of Erhai diatoms for effects of date and of first differences in ºdisorder (*x* axes) on first differences in biodiversity (*y* axes), showing estimates and 95% confidence intervals produced by R function 'gam'. (a) Pre-1966 lead-in partition for the full additive model: Biodiversity ~ s(Date) + s(°Disorder). Parametric linear regression on first differences in biodiversity revealed no significant effect of first differences in ºdisorder (t_{77} = 0.144, P = 0.886, model r^2-adjusted = 0.0) after removal of non-significant interaction with date, and date main effect. (b) 1966-201 pre-transition partition for the full additive model. Parametric linear regression revealed a strongly negative main effect of first differences in ºdisorder (r = -0.688, t_{18} = -4.019, P = 0.001, model r^2-adjusted = 0.444) after removal of non-significant interaction with date, and date main effect. (c) 2001-2009 transitioned partition, with 15 observations sufficing only for smoothing in individual models of each effect on biodiversity. Parametric linear regression revealed a positive main effect of date (t_{12} = 3.439, P = 0.004, model r^2-adjusted = 0.454) after removal of non-significant interaction and main effect of first differences in ºdisorder. Inspection of residuals for the best-fitting regression models indicated insufficient changes with the mean or deviations from normality to warrant data transformations or use of non-Gaussian error structures.

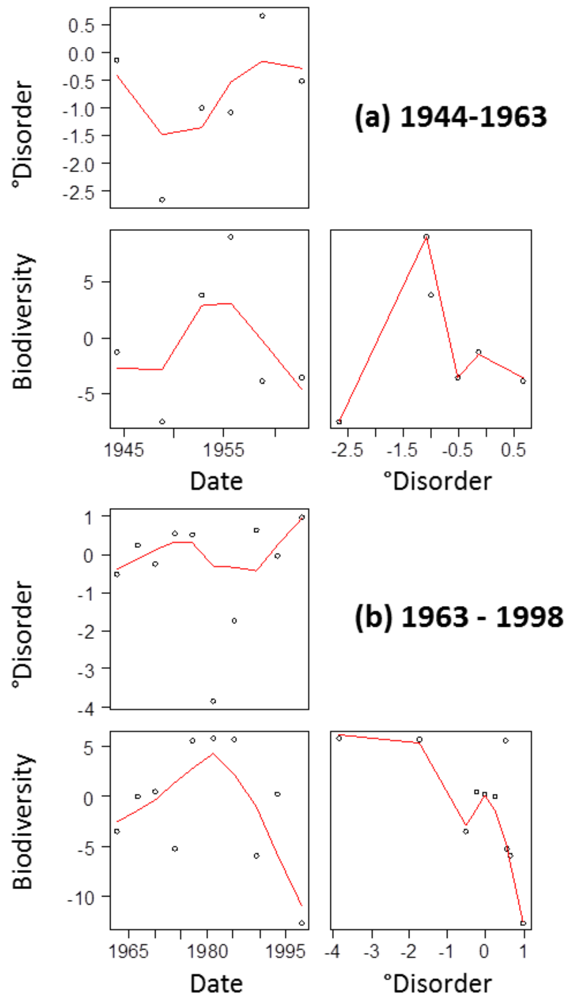

Fig. S5. Consolidated dataset of first differences from equal time intervals, Erhai diatoms. Pairwise correlations of date, ºdisorder, and biodiversity for (a) lead-in, (b) pre-transition
