# Supplementary material for: Early warning of critical transitions in biodiversity from compositional disorder
Source: Ecology. 2016 Nov 3;97(11):3079–90. doi: 10.1002/ecy.1558 (PMC6849621; doi:10.1002/ecy.1558)
Supplement: Supplementary file 3 [file ECY-97-3079-s003.pdf]

APPENDIX S3. Supplementary analyses of critical transitions

Supplementary evidence of break-points in the empirical time series was sought from detrended correspondence analysis (DCA) of the same datasets of species abundances through time as those used for main-text Fig. 3. DCA axis scores were computed with the 'Canoco' software package, using segment detrending methods on square-root transformed percentage abundances of species with down-weighting of rare species. DCA is a gradient analysis which clusters samples within orthogonal gradients. Here, we choose the two most important gradients (DCA axes 1 and 2) to identify possible break points from 1950 for the three lakes.

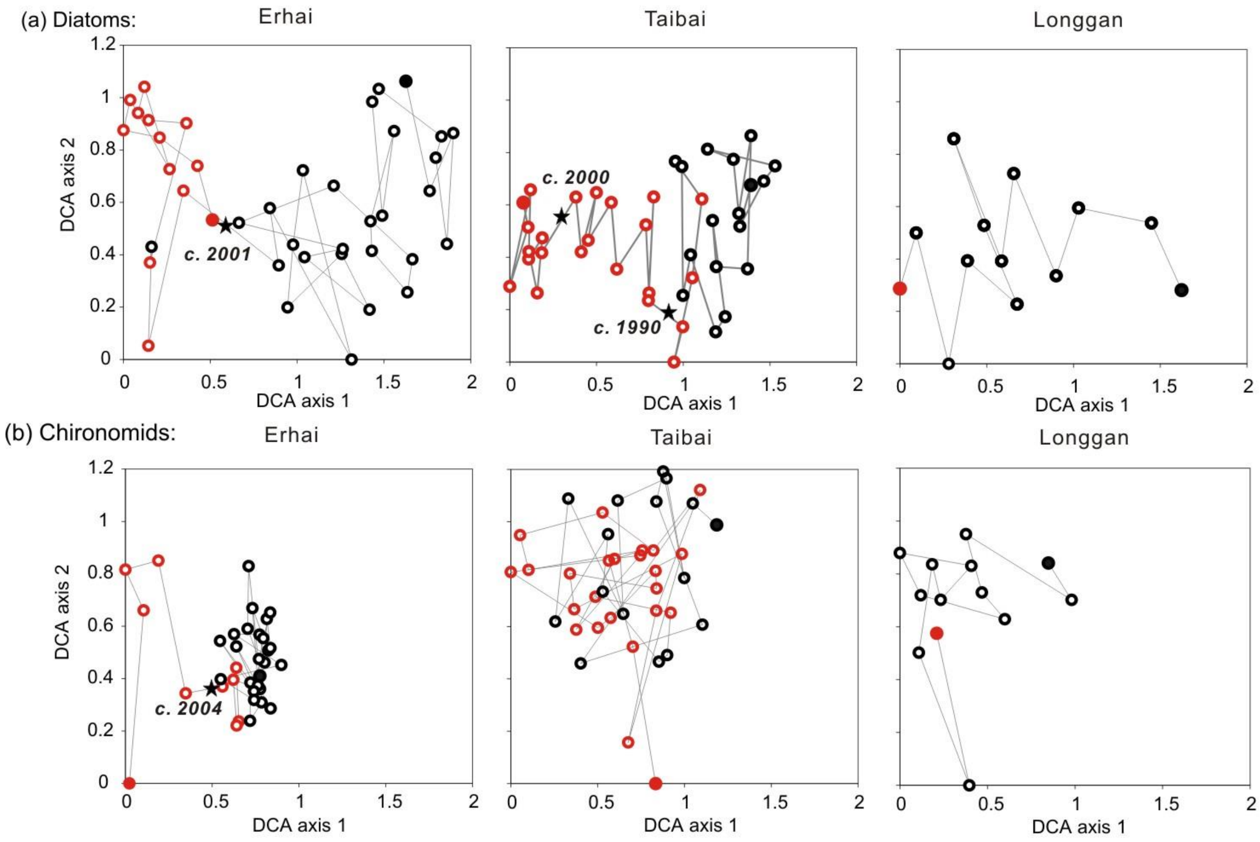

FIG. S1. Ordination of DCA in communities of diatom and chironomids. Lines join consecutive sections (dots) through each core, from 1950 (filled black dot) to 2006/7 (filled red dot). Red dots show sections post-2001 and post-1986 in Erhai and Taibai lakes respectively (cf hashed vertical lines in main-text Figs 3-4). Dated stars show approximate break points identified by the segregation of sequential points into discrete clusters.
