# Supplementary material for: Early warning of critical transitions in biodiversity from compositional disorder
Source: Ecology. 2016 Nov 3;97(11):3079–90. doi: 10.1002/ecy.1558 (PMC6849621; doi:10.1002/ecy.1558)
Supplement: Supplementary file 4 [file ECY-97-3079-s004.zip › VIGUIER_program-user-guide.pdf]

# A stochastic agent-based model of Lokta-Competition competition in diatoms community
# - User guide -

Clément VIGUIER

Version 1.0, September 2013

**Abstract**

This document is intended to the users of the program "A stochastic agent-based model of Lokta-Competition competition in diatoms community" [1] and associated functions. It will detail how to use this model to run customized simulations and comment the main and side functions. This program is design to simulate evolution of a diatoms community driven by Lokta-Volterra competition model. This stochastic model is a non spatially explicit agent-based model coded in Matlab[2]. For more details on the model please refer to my report and the following article of Doncaster and al.[3].

[1]Programmed with Matlab 7.8.0 (R2009a)

[2]This is a working version, it does not contain any control on parameters and arguments, errors can occur.

[3]Patrick C. Doncaster, Vashti Alonso Ch, Rong Wang, John Dearing, Enlou Zhang, Peter G. Langdon and James G. Dyke, *Early warning of a critical transition in biodiversity from community nestedness*,Unpublished

# Contents


**1 Preamble** **1**

**2 How to use the program** **2**
2.1 Use main script and repetitive script . . . . . 2
2.1.1 Run one simulation . . . . . 2
2.1.2 Run a sequence of simulations . . . . . 3
2.2 How to change parameters . . . . . 3

**3 How the program works** **6**
3.1 Program layout . . . . . 6
3.2 Detailed functions . . . . . 7
3.2.1 Initialisation . . . . . 7
3.2.2 Evolution of the ecosystem . . . . . 9
3.2.3 Display and analysis . . . . . 11
3.2.4 Secondary function . . . . . 12
3.3 Optimisation . . . . . 12

**A Using the program** **13**
A.1 Example of use . . . . . 13
A.1.1 Use the script `Ecosystem_2707` . . . . . 13
A.2 Script, functions and variables . . . . . 15
A.2.1 Scripts . . . . . 15
A.2.2 Functions . . . . . 24
A.2.3 Variables . . . . . 28

# Chapter 1

# Preamble

The following program has been designed to support the detection of a new type of Early Waarning Signal in the evolution of community's structure of diatoms.

This program is a stochastic simulation of the evolution of an ecosystem in a finish environment. The model proposed is based on a community of diatoms, but it can be adapted to any other ecosystem with strong competition. Any agent can be either a individual or a group of individuals depending on the ecosystem modelled. Each agent is affiliated to a species characterized by an intrinsic reproduction rate $c$. The intrinsic death rate is the same for all species but can change over time to simulate habitat degradation. Differences only in $c$ is sufficient ot defined different intrinsic growth rate between species. Moreover, each species have competition coefficient to determine impact imposed and given from and on other species during competition. Each agent have to settle in a patch to persist in the environment. The environment is modelled as an array of $K$ patches. Because the size of the environment is supposed to be finished, each patch can contain zero or one agent, avoiding possible stacking of agents, and leading to competition. The simulation starts with a particular number of species $n$ ($n = 30$), each with the same number of agents. At each time steps agents have a chance to reproduce and to die, and news species (from a meta-population of 150 species) can invade the environment and try to settled in a patch. The population run over a number of time after what results can be saved and analysed.

# Chapter 2

# How to use the program

This first chapter is intended for all users, unlike chapter 2 written for users which would like to understand how the program works and explore the code. I will explain you how to use the program to have the results of one simulation, but also how to run a sequence of simulations and how to change the parameters to set customized simulations.

## 2.1 Use main script and repetitive script

**First steps** This program is coded in Matlab (language easy to read, which does not require to be compiled, include visualization tools and allows optimisation by vectorization) so you need Matlab to be able to run this program. Extract the contain of the archive in a folder of your choice and change your Matlab current directory to this folder. You are now able to see all functions and scripts you can use.

### 2.1.1 Run one simulation

To run simulation write `Ecosystem_2707` in the command window.

**Display of results** Now the simulation is launched, and you can see some informations on your screen relative to the parameters. It could be interesting to have these informations to have the context of the results. Moreover, even if the script `Ecosystem_2707`use default parameters you can change them 2.2, and it is nice to see them for every simulation. Notice that you can chose to have no display changing the option `displayr` to `'none'`. Press any key to start the time evolution of the ecosystem.

After a few second (around 20 with default parameters) you should be able to see evolution of the population of the different species over time. Colours represent the properties of the species, the more red they are the higher their competitive ability is, the more green they are the higher their reproductive ability is high. You can easily differentiate keystone species (red-orange) from weed species (green) and canary species (yellow). Press any key to see the total population and biodiversity evolution, and once again to see variations is community composition. A brief summary is printed in command window.

You can also call the function `ecosystem_a` with or without arguments, but the default options are not the same to allow sequenced calls, see subsection below.

**Save** The script contain a save option and will ask you (by default) if you want to save the data. The compact save just save biodiversity and presence-absence matrix, instead of the complete mode which save data (population of each species, flux and number of agent and species of each group) in separated files and all variables in a file `.mat`.

### 2.1.2 Run a sequence of simulations

Now you have done your first simulation and you want to see more, you want to analyse the data of a lot of simulations. You have to run the script called `Repeat_simulation`, which will call the function `ecosystem_a`with particular parameters. The function `ecosystem_a` does the same job that `Ecosystem_2707`, but can have input arguments and gives a formatted array of results interpreted by `Repeat_simulation` script.

**Modify the script** Because this script is a working version it is not user friendly, you have to open the source file in Matlab editor (or other editor such as Notepad) and modify the variables `mode` and `val`. Each value of the vector `mode` is a parameter you want to change (see section2.2 bellow for details) and the vector `val` contains the value you want for each parameter. Notice that if you want to test two value of the same parameter, its label has be in the `mode` vector twice. Both vectors have to be consistent (same length, same order, good type).

**Results** Because you run each simulation (defined by a parameter set) many times, you wont want to see evolution of population over time (you are able to do so changing default parameter of display in `ecosystem_a`), so all results are mean and standard deviations of interesting values as biodiversity, total population, number of agents of each group, in average or at the end of the simulation. You have also a Species Abundance Distribution diagram for each set of parameters (see function `SAD`).

**Save** By default the data are not saved by the function `ecosystem_a` but you can change the related options to allow save (2.2). The variables containing the results of sequences of simulations are automatically saved.

## 2.2 How to change parameters

There are two ways to change your parameters, the first one is to use optional arguments of `ecosystem_a` function, the other one is to change directly the parameters in the code.

**Chose the good arguments** When you just want to change simple parameter such as the time, number of starting species, size of the environment, you will use the function `ecosystem_a` with input arguments. These arguments are the same that `Repeat_simulation` script uses for sequential simulations. For now the following arguments are possible :

$K$ (`K`) the size of the environment $K \in ]K_{min}; K_{max}[$ where $K_{min} > p * n$ where $n$ is the number of starting species (understand species with a non-zero population at the beginning of the simulation) and $p$ is the number of agents for each of these species. Notice that if $K$ is too big the simulation will take lot of time and resources to be performed (errors can occur), so $K_{max}$ is depending on your computer and your time.

$t$ (`time`) the time of the simulation, $t \in \mathbb{R}_{+*}$ Same remark can be make for the performance limit.

$D$ (deg) $0$ for no habitat degradation, $1$ for a fluctuating habitat degradation (see 3.2.1 for more details), $2$ for a two phases degradation and $3$ for a linear degradation and recovery.

$d$ (ddef) is the default value of $d$.

$i$ (invform) $0$ for a constant rate of incoming species, $1$ for a fluctuating rate of invasions (see 3.2.1 for more details).

$n$ (n) number of starting species, $n \in ]1; N[$, where $N$ is the number of species in the meta-population, be careful that $p * n < K$ where $p$ is the number of agents for each starting species.

$s$ (trade-of) $0$ for an linear trade-off, any other option will set the trade-off as the default one (sigmoidal).

$N$ [1](N) the number of species in meta-population $N$, $N >= n$. If $N$ is too big, computation of alpha matrix and trade-off can be long.

$b$ (beta) for $\beta$, it is the slope of the sigmoïdal trade-off. It has to be negative.

$P$ (phi) for $\Phi$, it is the level of randomization of competition, if $\Phi = 0$ competition is fully deterministic, if $\Phi = 1$ competition is completely random.

To change several parameters at the same time you have to write the different modes in a first vector and the values in second one. These vectors need to have the same length. The script Repeat_simulation use a matrix of modes and a matrix of values. Each couple of rows $mode_i$ and $val_i$ is the couple of arguments used by the function ecosystem_a to run simulation with the set of parameters $i$. Be careful to have a matrix of rows with the same number of columns, to avoid concatenation problems fill the empty spaces with $0$. See chapter A.1 for more details.

**Modify the code** When you want to perform all your simulations with a particular set of parameters or to test a completely different set than the default one, you have to modify the code directly in editor. Open the file Ecosystem_2707 or ecosystem_a and change the value of the parameters you are interested in. Values have to be consistent with the parameter meaning and others parameters values (errors can occur, i.e. too many starting species in a small environment) . You should be sure you have a unmodified version of the program when you modify manually the parameters.

**What parameters and options change** In addition to the parameters listed in previous paragraph 2.2 (which can also been changed directly in the code), some parameters or options have to be changed in the script Ecosystem_2707 or function ecosystem_a.

Here there is some interesting parameters you should want to be able to change :

$c_{min}$ and $c_{max}$ are the minimum and maximum value of $c$. These values directly the slope of the linear trade-off ($\frac{1}{c_{max}-c_{min}}$).

$p$ or $p_{max}$ controls the number of agents for the $n$ starting species (or respectively the maximum number of agents for incoming species).

There is also some options available to fit the program with the use you have of it :

[1]Cannot be tested in current version of Repeat_simulation script. Use only directly with ecosystem_a.

**saveopt** manual if you want the program to ask you at the end of each simulation if you want to save your data (default value in Ecosystem_2707), auto if you want an automatic save (compact save by default, change variable w2save to 2 in the part save data) or none if you do not want to save (default value in ecosystem_a).

**displayr** complete if you want to see all diagrams and results (default value in Ecosystem_2707), compact if you do not want diagrams, and none for no display (default value in ecosystem_a).

**firstDsave** is the first time-step to be record, useful if you do not want to save first hundreds of time-steps needed to stabilize the system.

**reverserows** 1 to reverse order of row, useful to match the format of save with the format of data you use (e.g. data from sediment core with a reversed time-line).

# Chapter 3

# How the program works

This chapter is intended to users who want to know how this program works. A part of this chapter is dedicated to explain the code and detail techniques used, you should need some programming knowledges to understand this part.

## 3.1 Program layout

The program is organized in three big parts, first initialization which set up all variables and parameters, simulation of the ecosystem evolution over time and finally extraction, display and save of data. These three components are details in the following figure.

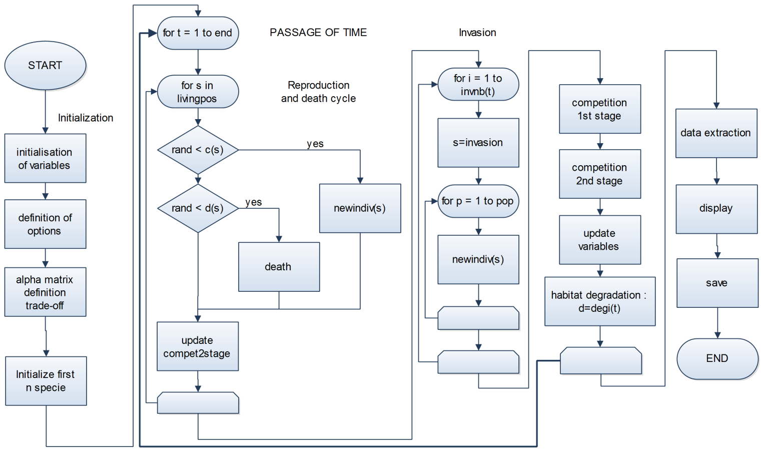


Figure 3.1: Functional flowchart of `Ecosystem_2707` and `ecosystem_a`

## 3.2 Detailed functions

### 3.2.1 Initialisation

Here are detailed functions used in initialisation or related to this part of the program.

**Influx-Outflux** Lake ecosystem are related to other lakes and rivers in a complex network, called drainage basin, characterised by the meta-population of 150 different species. In this program only evolution of the lake system connected to this network is modelled. In this contest, the lake ecosystem can be considered as a chemostat with a input and an output. So the global influx is the flux of incoming from meta-population and previously absent in the local system.

**Invasion and degradation** In this chemostat the global influx is the flux of incoming species from meta-population and previously absent in the local system. The global outflux is the ensemble species which disappear at each iteration. If the influx can be set up by modifying the variable `invnb`, the global outflux is drive by changing in $d$ value, equals for all species, so the value of $d$ (variable `degi`) over time is considered as the global outflux in the program. As said before (2.2), three different regimes of degradation are available. The first is simulation of a fluctuating environment around an average value, this value can be modified using the input arguments. The second regime starts the same way but after a while, fluctuations mean value increases. The third one is a linear up and down of $d$ value. As for the first regime, in the next two the reference value of $d$ (variable `ddef`) can be changed by option $d$. The amplitude of fluctuations and their period can be changed by changing inputs arguments of function `flux` and `flux2ph`, and variable `T` for the period.

**Flux measure** The global influx and outflux are tools to simulate changing in environment (e.g. local degradation of habitat is simulate increasing the outlfux with a constant influx). But, flux in species of the three different types can be measured and give informations on the system. This measure is provided by direct measure over time of the number of new species of each type after the called of the function `invason`.

**Trade-off between competitive ability and intrinsic growth rate** To avoid “super-species” a trade-off between intrinsic growth rate and competitive ability has to be set up. Because building of alpha-matrix needs a lot of control to increase the range of value of competitive ability, and because the dependence between rows and columns are strong, this matrix is build first. Then, the growth rate is determine as a function of the competitive ability.

**Building of alpha-matrix** Alpha-matrix contains all competition coefficients $\alpha_{ij}$ between all possible couples bet species $i$ and $j$. For each species $i$, the attack ability is defined as the mean of the column $\alpha_{c_i} = \bar{\alpha_{.i}}$, and the vulnerability as the mean of the row $\alpha_{r_i} = \bar{\alpha_{i.}}$. A global competitive ability can be defined as a function of these two values. This value, noted $\bar{\alpha_i}$ has to be high for a high attack ability and a low vulnerability. The $\bar{\alpha_i}$, noted `alphabar` in the program, is so calculated as described below by the function `inv_norm`, after a rescaling of $\alpha_{c_i}$ and $\alpha_{r_i}$. This rescaling gives $\alpha_{c_i} = 1$ to the best attacking species (respectively $\alpha_{c_i} = 0$ to the worst attacking species), and $\alpha_{r_i} = 0$ to the best defender (and $\alpha_{r_i} = 1$ to the most vulnerable).

$$\bar{\alpha_i} = \frac{1 - (\alpha_{r_i} - \alpha_{c_i})}{2}$$

First intuitive solution to fill the alpha-matrix is to create a random square matrix, but if this solution give variability in coefficients $\alpha_{ij}$, it does not provide the variety in global behaviours. That

means it is difficult to distinct attacking species from defensive species, because mean of rows and columns, and so $\alpha_c$ and $\alpha_r$, are all concentrated around 0.5. To avoid this, a gradient is imposed on columns to impose a gradient of attacking ability $\alpha_c$. The range of this gradient, noted `hd`, is less than 1 to allow variability on rows. Minimum value of the rows is chosen between $0$ and $1 - hd$ under a non-uniform random function[1]. If affect one colour to each value of this matrix, we can visualize it as shown in 3.2.1, where the last row and the last column are representation of means on columns and rows.

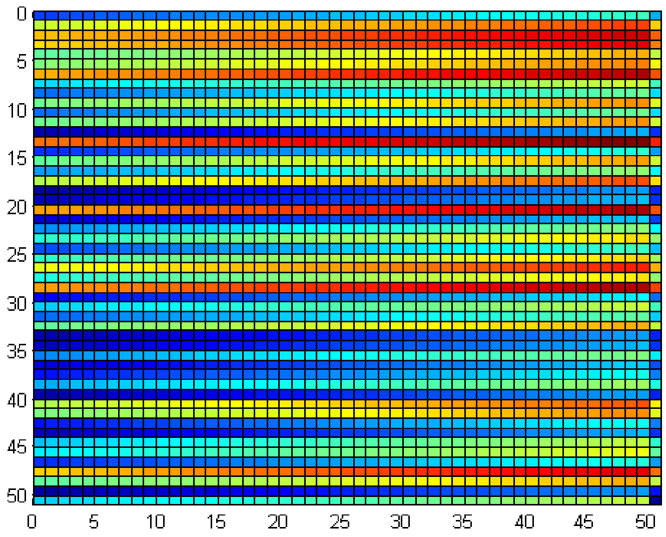


Figure 3.2: Representation of alpha matrix

Example of representation with colours of alpha matrix (`alph`) for 50 species. Last row and last column are respectively means on columns and rows. Columns' gradient is observable on the last row, although randomization of rows is represented by non linear variations in last column.

Here below the code to build the alpha matrix.

```
%Alpha (called alph) matrix : competitive ability (between 0 and 1) :
%competition coefficients for couple of two species,
%alpha(i,j) is the ability of j to beat i

hd=0.5;            %horizontal deviation, define the range of alpha_c
a=1-hd;            %possible vertiacl range
for i=1:N
    r=urand*a;     %randomisation of alpha_r, non uniform to enlarge range
    for j=1:N
        p=(2*(j/N-0.5)).^2;
        alph(i,j)=r+(hd/N)*j;
    end
    alph(i,i)=1;
end
```

[1]See random function in for more details 3.2.4.

**Trade-off functions** Several trade-off can be tested to give more flexibility to the program. First trade-off, and more intuitive, is the linear trade-off, provided by function `tradeofalpha`. This function caps the $c$ value by the following function :

$$c_{ceiling} = c_{min} + (c_{max-c_{min}})(1 - \bar{\alpha})$$

The second one is the sigmoidal trade-off, which gives more power to keystone species. This is a ceiling by classic sigmoid, imposed on $c$ by the function `tradeofsig`. $c_{min}$ permits to translate the trade-off, and $c_{max}$ avoids an theoretically infinite value possible for $\bar{\alpha}$ near to 0, but it does not change the sigmoid position as $c_{min}$ does. The slope $\beta$ of this trade-off is an important parameter and can be set by the dedicated option $B$. The following figure presents the sigmoid for different value of $\beta$.

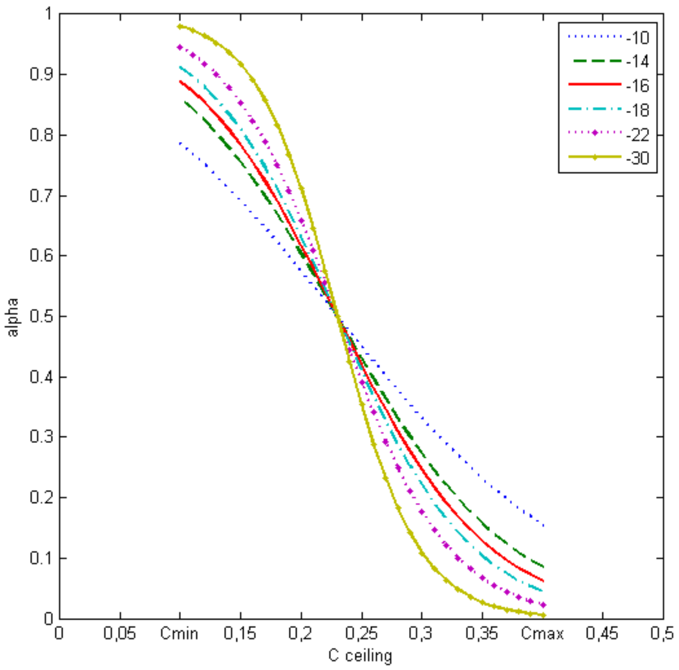


Figure 3.3: Different shapes of sigmoid trade-off for different value of $\beta$

The last function is inverse function of a sigmoid.

### 3.2.2 Evolution of the ecosystem

**Reproduction, death and invasion** The reproduction and death are simulated by stochastic events. For each living individual two random numbers are generated and compared to respectively $c$ and $d$ of the species, if the first is not greater than $c$ a new propagule is added to competition list and if the second is less than $d$ the agent is removed.

Invasion is simulated by the function `invasion` which pick randomly a species in the list of extinct species, then between 1 and 5 new propagules are added to competition list. The variable `invnb` imposes the number of iteration of this step previously described.

**Competition** The competition is the part of the simulation which take the most of the execution time (around $30\%$) and is one of the more complex to optimize. Because competition is asymmetric, some agents will defence their patch, others will try to invade it, the competition is divided in two stages. The first one, based on relative competitive ability, permits to incoming agents who want the same patch to decide between them the one which will compete against a resident agent if there is one. The second stage decides between incoming winners of the first stage and pre-established agents. Because there are roles of defender and attacker in this stage, list of the competitors has to contain this information, this information in order of agent in the array. Indeed, the pre-established agents are included in the array before incoming agents, so after sorting the array by patch to group competitors, in case of competition (two successive agents claiming for the same patch), the first one of two will always be an established agent, and so a defender. Competition algorithm is detailed in the flowchart below.

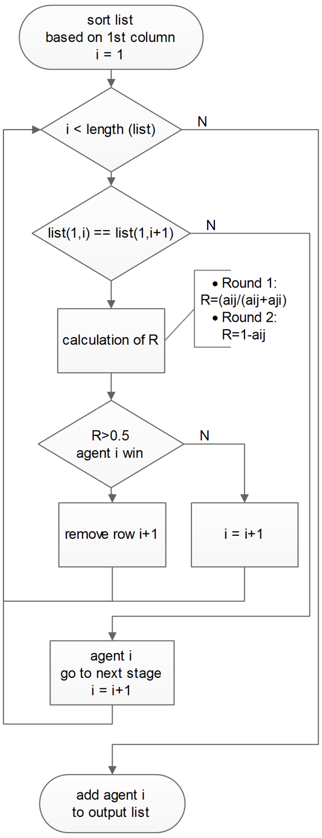


Figure 3.4: Flowchart of competition algorithm

The same function `compet` deals with the two stages, it changes the calculus of the probability of victory for the agent $i$ against agent $j$ ($R_1$ for the first stage and $R_2$ for the second stage) according to the parameter `mode`. The probability are described below:

$$R_1 = \frac{\alpha_{ij}}{\alpha_{ij} + \alpha_{ji}}$$
$$R_2 = 1 - \alpha_{ij}$$
$$P(i\ win) = P([R(1-\Phi) + \Phi rand(0:1)] > 0.5)$$

The function `compet` has the variable `win` as output argument, this variable contains the list of winning agent after competition.

**Habitat degradation** The habitat degradation is simmulated by changes in $d$ value for all species. At the end of each time iteration, the $d$ value is updated with the corresponding value of variable `degi` described earlier. Depending of the display mode, the program plots the degradation intensity over time.

### 3.2.3 Display and analysis

In the following paragraphs different plots display are detailed.

**Population evolution** At the end of the simulation, the evolution of the population of species over time is display. The program uses the plot function `surf` because it is by far the most efficient function in Matlab to display 3D of this dimension. Depending on your computer's performances, the limit on the number of time-steps allowing a good visualization could change. In addition, this function permits to change colours of the surface. Although, the colour of the faces reflects the properties of the species. The green fraction represents the reproductive rate, the red represents the competitive ability and the yellow the weakness for these two abilities. So, the species represented green are 'weed' species, the red are the 'keystone' species and the yellow are 'canary' species. White is species with 0 agent. Changing the view of the plot permits to see species' dynamics hidden by numerous species, but if you watch the graph with an opposite point of view from the default one, colours lose their meaning.

**Total population and biodiversity** To follow evolution of simulation total number of agents is plotted at the end of the simulation. To give an idea of diversity, the abundance of the most numerous species is plotted on the same graph. Under that graph, biodiversity and number of species present in the environment are also plotted.

**Structure analysis** All species are defined by $c$ and $\alpha$, these values permit to distinct three functional groups: keystones, weeds and canaries. After trade-off, all species are separated in these groups base on both $c$ and $\alpha$ value. Because it is mathematically difficult to separate a sigmoïd in three equal parts, and because in the first steps of development the trade-off was linear, the separation within groups is based on angle of vector $\vec{v_i}(c, \alpha)$. If its norm is under $\sqrt{\frac{2}{3\pi}}$ the species is considered as a canary species, otherwise the species is counted as a weed if its angle is under $\frac{\pi}{4}$ and as a keystone if its angle is greater than this threshold. This discrimination is done after rescaling, avoiding concentration in only one group, but you have to keep in mind that this functional distinction is relative to other species of the community. Number of agents and number of species for each group are respectively plots in two different plots.

### 3.2.4 Secondary function

**Random functions** Because of the dependence between rows and column in the alpha matrix, and because $\bar{\alpha}$ is calculated from means, manipulation of coefficient of alpha matrix by random function was necesary. Indeed, mean of uniform random function drives all values of $\bar{\alpha}$ around 0.5 and create community very even. In a way to analyse function of dominant competitors on fast reproducer, the difference had to be greater, so I use custom random functions.

**Parabolic random function: `urand`** This function give random number according to parabolic distribution. This parabola is defined by the following function, where $a$ is the depth of the parabola.

$$r = a((2x-1)^2 - 1) + 1, \ x \in [0:1]$$

Method of rejection used to get this distribution, it can be slow for a high number of random numbers but the method by inversion cannot be used because parabolic functions are not monotonous.

**Others random functions** Other functions as `srand` (sigmoidal distribution) or uniform Matlab function `rand` can be used in this program. You only have to change it in the alpha matrix construction.

**Save** The function `save_file(mat,name,OPTnb_rows,OPTnb_col)` create a text file containing coefficients of matrix `mat` named with the content of variable `name` (it must be a string finishing whit .txt). You can imposed the numbering of rows and/or columns thanks to the two last arguments.

## 3.3 Optimisation

Even if this model is applied to small ecosystems, optimization allows you to define customized parameters and run a simulation in less than a minute, it also allows to run sequences of hundreds of simulations in a few hours. In order to keep a reasonable execution time I mainly used two techniques.

**Discontinuous time line** First technique is to avoid automatic updates of variables, some as `pres` (and `pres`) can be easily updated massively with deductive calculus on matrix (very effective in Matlab), this way : `pres=logical(speciespop)`. A deductive approach also permits to determine variations in each group (keystones, weeds and canaries) at the end of the time loop (see `fluxmeasure.m`).

**Vectorization** The strength of programming in Matlab is used of matrix transformation and operation, because it is design to work with matrix. Vectorization of program consist in transforming operations made in a loop in operations on matrix to limit the cost of loops use.

# Appendix A

# Using the program

## A.1 Example of use

The current section shows an example of use of the different parts of the program. Because can incur some modifications, the screen-shots presented in this section can be different from what you see with your version of the program.

### A.1.1 Use the script `Ecosystem_2707`

Open Matlab and change the current directory to the folder containing the program's files. Type `Ecosystem_2707`in the command window and press 'Enter'

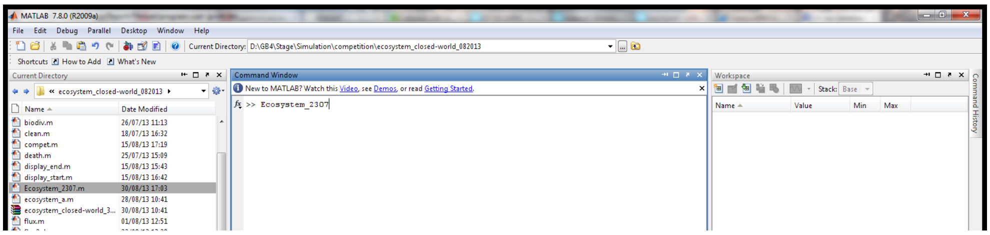


A plot of $c$ and $\alpha$ appears if the option display is set to 'complete' (default value)
Press any key to start simulation.
At the end of the simulation, graph of population evolution per species is displayed (depending of display mode).

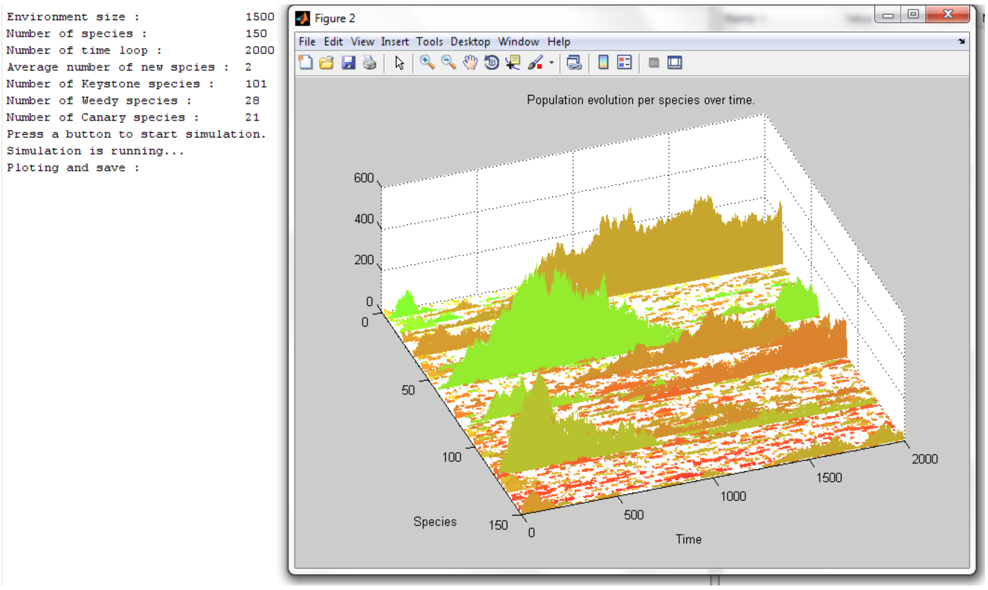


Press any to display the next graphs: population and biodiversity evolution, and structure evolution.

Finally a brief summary of simulation is printed on the screen and you can save the results in text files. Enter 1 for a short save (presence matrix and biodiversity over time), 2 for a complete save and 0 for no save, then press 'Enter'. Enter the base name of your files between simple quotation marks (`'example_of_use'` in the example).

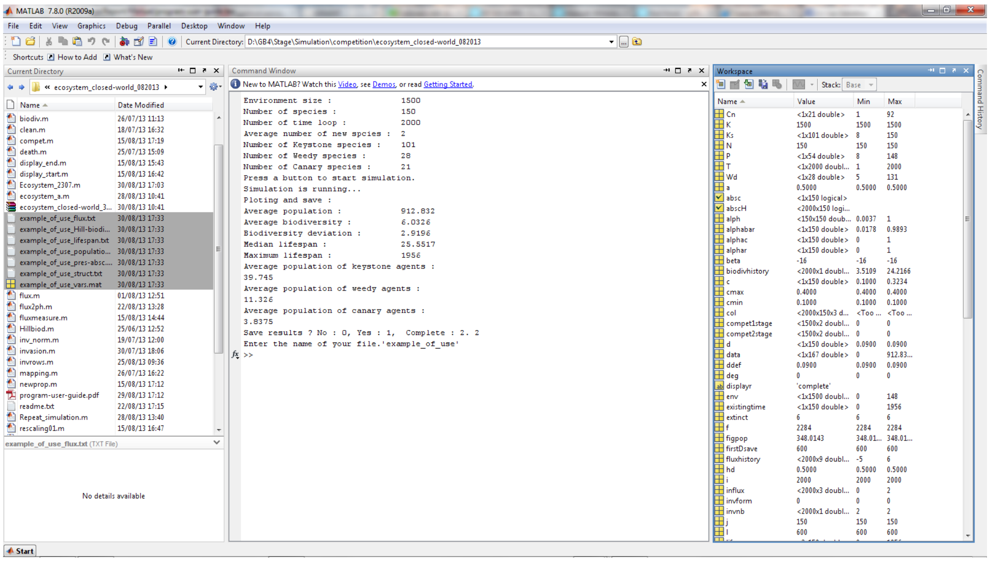


Your files are created in the current folder. Notice that you can access to all variables conserved in the Workspace after the end of the script. All text files can be opened in Matlab or in your favourite editor.

## A.2 Script, functions and variables

### A.2.1 Scripts

Find in this section the main script and functions.

The first script in the script `Ecosystem_2707`, the function `ecosystem_a` is not presented here. The script `Repeat_simulation` follow.

```
%%%%%%%%%%%%%%%%%%%%%%%%%%%%%%%%%%%%%%%%%%%%%%%%%%%%%%%%%%%%%%%%%%%%%%%%%%%%
%%%          Ecosystem simulation based on Lokta-Voltera                 %%%
%%%                     competition model                                %%%
%%%%%%%%%%%%%%%%%%%%%%%%%%%%%%%%%%%%%%%%%%%%%%%%%%%%%%%%%%%%%%%%%%%%%%%%%%%%

%Simulation of an open ecosystem colonized by species of diatomes
%from a non evolutive metapopulation.

%This is a script version allowing you to study all variables after the
%simulation. If you want to run a lot of simulation please use
%ecosystem_a.m function.

%created the 23th of July (2013)
%last update 15/08/13


%this version is a working version, it does not contain control of
%variables' consistency, errors can occur with different values than
%default values


clear all
clearvars -global
clc
format compact



%DECLARATION OF GLOBAL VARIABLES : ________________________________________

%Running variables : ______________________________________________________

global K                %size of the environment
global env              %environment : array of 1 row and K columns
global N                %numer of species in metapopulation
global sp               %list of species in metapopulation
global alph             %matrix of competion coefficients
global alphar           %mean of rows of alph matrix, 1-defensive ability
global alphac           %mean of columns, attacking ability
global d                %death rate
global cmin             %minimun growth/colonization rate
global cmax             %maximal growth rate for linear trade-of
global pres             %logical matrix of presence of each species
global absc             %non(pres)
global compet1stage     %list of new propagules and cell/patch claimed
global speciespop       %population of each species

%__________________________________________________________________________

%INITIALIZATION : _________________________________________________________

```

```
%Running variables and parameters setting : ---------------------------------

%These following values are default values but can be easily changed for a
%customized use of this script (be careful to keep a unmodified version of
%this script)

K=1500;                 %size of the environment
time=2000;              %number of time step
N=150;                  %size of the metapopulation
n=20;                   %starting number of species
cmax=0.4;               %maximum growth rate
cmin=0.1;               %minimal growth rate
beta=-18;               %slope of sigmodal trade-of
ddef=0.09;              %default vallue of d
d=zeros(1,N)+ddef;
invform=0;              %flux on invasion (0: constant 1: fulctutaion)
invnb=ones(time,1)*2;   %vector of number invasive species (mode 0)
deg=2;                  %default type of degradation (0: none, 1:global)
sens=ones(1,N);         %sensitivity to degradation/toxicity
tradeof='sigmoidal';    %trade-of form : -sigmoidal, -linear
phi=0.4;
env=zeros(1,K);         %initialisation of the environement
sp=(1:N);               %list of species number
pres=false(1,N);        %initialisation of the presence matrix
absc=true(1,N);         %initialisation of the abscence matrix
compet1stage=[];        %initialisation of competition lists
compet2stage=[];

if invform≠0
    %vector of number of invaders over time
    invnb=flux(0,4,2,round(0.3*time),round(0.6*time),...
        round(0.05*time),time);
end

%vector of degration impact (d=degi.*sens)
%set deg=0 to avoid degradation.
T=50;
if deg==1
    %- 1 fluctuating phase
    degi=flux(0.09,0.16,ddef,round(0.3*time),round(0.6*time),...
        T,time);

elseif deg==2
    %- 2 phases : fluctuating around ddef, fluctuating and increasing
    degi=flux2ph(0.04,ddef,0.005,round(0.4.*time),round(0.7.*time)...
        ,time,T,time);

elseif deg==3
    %- increasing and decreasing linear phases between values 'low' & 'top'
    degi=zeros(1,time)+ddef;
    phase1=(round(0.15.*time):round(0.5.*time));
    phase2=(round(0.5.*time):round(0.85.*time));
    low=ddef; top=0.21;
    degi(phase1)=(low:(top-low)/(length(phase1)-1):top);
    degi(phase2)=(top:-(top-low)/(length(phase2)-1):low);
end

%Recording variables : -----------------------------------------------------

speciespop=zeros(1,N);          %population of each species over time
pophistory=zeros(time,N);       %reccord of population evolution
preshistory=zeros(time,N);      %presence/absence matrix over time
```

```
influx=zeros(time,3);            %number of invaders species of each type
existingtime=zeros(1,N);         %time since species have been present
lifespan=zeros(2,N);             %1st line : mean lifespan, 2nd : weigth

%Options : ----------------------------------------------------------------
saveopt='manual';       %'auto' or 'manual' save (or 'none')
%'auto' gives an automatic name and saves biodiversity and presence matrix
displayr='complete';    %display results : 'complete', 'compact' or 'none'
%complete shows all diagrams, compact is limited to summaries.
firstDsave=600;           %day of beginnning of saving (stabilization
%phase finished around 600)
reverserows=1;          %reverse order of rows (=1) or not (=0)

%_______________________________________________________________________

%CREATION OF THE WORLD : __________________________________________________

%Initialization of species specific variables : ---------------------------

%Alpha (called alph) matrix : competitive ability (between 0 and 1) :
%competition coefficients for couple of two species,
%alpha(i,j) is the ability of j to beat i

hd=0.5;           %horizontal deviation, define the range of alpha_c
a=1-hd;           %possible vertiacl range
for i=1:N
    r=urand*a;    %randomisation of alpha_r, non uniform to enlarge range
    for j=1:N
        p=(2*(j/N-0.5)).^2;
        alph(i,j)=r+(hd/N)*j;
    end
    alph(i,i)=1;
end

% mapping(alph);

alphac=rescaling01(mean(alph,1));         %means of attacking ability
alphar=rescaling01(mean(alph,2)');        %means of 1-defence ability
alphabar=inv_norm(alphar,alphac);         %global competitive ability

%Intrinsic growth/colonization rate (between 0 and 1) :
if strcmp(tradeof,'linear')               %linear trade-of between cmin-cmax
    c=tradeofalpha(alphabar);
elseif strcmp(tradeof,'inverse')          %sigmoid inversed
    c=tradeofinv(alphabar,beta);
else
    c=tradeofsig(alphabar,beta);          %sigmodal trade-of (default)
end

%structure ananlysis based on c and alphabar :
[Ks,Wd,Cn]=structure(sp,alphabar,c);

%brief summary of condition
if strcmp(displayr,'complete') || strcmp(displayr,'compact')
    display_start(N,K,time,invnb,Ks,Wd,Cn)
end

%display alphabar/c trade of
if strcmp(displayr,'complete')
    subplot(111)
    plot(c(Ks),alphabar(Ks),'^k',c(Wd),alphabar(Wd),'ok',...
        c(Cn),alphabar(Cn),'*k')
```

```
    axis([0 1 0 1])
    xlabel('c')
    ylabel('alpha')
    disp('Press a button to start simulation.')
    pause
end

disp('Simulation is running...')

%Peopling of the world with the first n species : -------------------------
l=0;
for i=0:n-1
    s=invasion;                         %return the index of a species
    p=30;                               %initial population per species
    for j=1:p
        speciespop(s)=speciespop(s)+1;  %update population
        l=l+1;                          %invade the next cell
        env(l)=s;
    end
end
living=env(env>0);       %living agents
pos=(1:K);
livingpos=pos(env>0);    %positions occupied by a living agents

pop0=speciespop;         %record initial population
%___________________________________________________________________________

%TIME LOOP : _______________________________________________________________

%%%%%%%%%%%%%%%%%%%%%%%%%%%%%%%%%%%%%%%%%%%%%%%%%%%%%%%%%%%%%%%%%%%%%%%%%%%
for t=1:time %%%%%%%%%%%%%%%%%%%%%%%%%%%%%%%%%%%%%%%%%%%%%%%%%%%%%%%%%%%%%%
   for i=1:length(living)
       s=living(i);
       %each living agent has a chance to reproduce and to die

       %reproduction
       if rand < c(s)
           [speciespop,compet1stage]=newprop(s,K,speciespop,compet1stage);
       end
       %death
       if rand < d(s)
           p=livingpos(i);
           env(p)=0;
       %defence of its cell/patch
       else
           f=size(compet2stage,1);
           p=livingpos(i);
           compet2stage(f+1,1:2)=[p,s];
       end
   end

   %update speciespop, pres and absc
   %do not need an continuous update of these variables
   for i=1:N
       s=sp(i);
       speciespop(s)=sum(env==s);
   end
   pres=logical(speciespop);
   absc=¬pres;

   %Invasion : ____________________________________________________________
   for i=1:invnb(t)
```

```
        s=invasion;
        if s≠0;
            %randomize number of invaders agents
            pmax=5;
            p=round(1+(pmax-1)*rand);
            for j=1:p
                [speciespop,compet1stage]=newprop(s,K,speciespop,...
                    compet1stage);
            end
            %influx recording
            if Ks(Ks==s)==s
                influx(t,1)=influx(t,1)+1;
            elseif Wd(Wd==s)==s
                influx(t,2)=influx(t,2)+1;
            else
                influx(t,3)=influx(t,3)+1;
            end
        end
    end

    %Competition : ------------------------------------------------------------
    %1st stage of competition between news propagules from reproduction or
    %invasion, based on relative attacking capacity

    stage2=compet(compet1stage,1,alph,phi);

    %2nd between settled individuals and winners from 1st stage, there can't
    %be more than two competitors for the same patch/cell. Elders must be
    %first in the array to have the good competition coefficients
    compet2stage=[compet2stage;stage2];
    winners=compet(compet2stage,2,alph,phi);
    for w=1:size(winners,1)
        env(winners(w,1))=winners(w,2);
    end
    %clear the competition lists
    compet1stage=zeros(K,2);
    compet2stage=zeros(K,2);

    %Update and record : ------------------------------------------------------

    %update living position list to make reproduction and death test only in
    %these cells
    living=env(env>0);
    pos=(1:K);
    livingpos=pos(env>0);

    %update speciespop, pres and absc
    for i=1:N
        s=sp(i);
        speciespop(s)=sum(env==s);
    end
    pres=logical(speciespop);
    absc=¬pres;

    %record :
    pophistory(t,:)=speciespop;
    preshistory(t,:)=pres;

    %update existingtime - lifespan
    extinct=find(absc&logical(existingtime));
    existingtime=existingtime+pres;
    lifespan(1,extinct)=(lifespan(1,extinct).*lifespan(2,extinct)...
```

```
        +existingtime(extinct))./(lifespan(2,extinct)+1);
    lifespan(2,extinct)=lifespan(2,extinct)+1;
    existingtime(extinct)=0;

    %Environment degradation : -------------------------------------------
    if deg≠0
        d(sp)=degi(t).*sens(sp);
    end

end%%%%%%%%%%%%%%%%%%%%%%%%%%%%%%%%%%%%%%%%%%%%%%%%%%%%%%%%%%%%%%%%%%%%%%%%%%%%%
%%%%%%%%%%%%%%%%%%%%%%%%%%%%%%%%%%%%%%%%%%%%%%%%%%%%%%%%%%%%%%%%%%%%%%%%%%%%%%%%

%__________________________________________________________________________

%LAST STEP AND DATA EXTRACTION : __________________________________________

%Calculation of "species lifespan" for non extinct species
P=find(pres);
lifespan(1,P)=((lifespan(1,P).*lifespan(2,P))+existingtime(P))...
    ./(lifespan(2,P)+1);

%Data extraction : --------------------------------------------------------
T=(1:time);

%Hill's biodiversity :
biodivhistory=biodiv(pophistory);

%Number of species and maximum population over time :
sphistory=sum(preshistory,2);   %number of species at each time step
totalpoph=sum(pophistory,2);    %total population at each time step
maxpoph=max(pophistory,[],2);   %population of the most numerous species

%Influx-Outflux-Net flux
netflux=fluxmeasure(preshistory,pop0,Ks,Wd,Cn);
outflux=influx-netflux;
fluxhistory=[influx,outflux,netflux];

%structure analysis (nb of canaris, weeds and keystones) :
popKs=sum(pophistory(:,Ks),2);
popWd=sum(pophistory(:,Wd),2);
popCn=sum(pophistory(:,Cn),2);
nbKs=sum(preshistory(:,Ks),2);
nbWd=sum(preshistory(:,Wd),2);
nbCn=sum(preshistory(:,Cn),2);
str=[nbKs,popKs,nbWd,popWd,nbCn,popCn];
%__________________________________________________________________________

%EXPORT DATA : _____________________________________________________________

data=[mean(biodivhistory),mean(totalpoph),mean(sphistory),mean(str,1)...
    totalpoph(time),sphistory(time),str(time,:),pophistory(time,:)];
%__________________________________________________________________________

%SAVE AND DISPLAY : ________________________________________________________

%Display : -----------------------------------------------------------------

if strcmp(displayr,'complete')
    if deg≠0
        plot(degi,'k.')
    end
    title('Variations of ''d'' over time.')
```

```

    figure
    disp('Ploting and save :')
    %plot species' populatioon evolution
    %Definition of colors
    sr=0.25;
    sg=0.12;
    sb=0.18;
    col(:,:,1)=(sr+1-(1-sr).*c(ones(1,time),:)./max(c));
    col(:,:,2)=(sg+1-(1-sg).*alphabar(ones(1,time),:));
    col(:,:,3)=sb;
    %make 0 values white
    abscH=¬(preshistory);
    for i=1:size(col,1)
        for j=1:size(col,2)
            if abscH(i,j)==1;
                col(i,j,:)=ones(1,1,3);
            end
        end
    end
    %plot
    figpop=surf(pophistory,col);
    shading flat
    view(70,60)
    title('Evolution of number of agent per species over time.')
    ylabel('Time')
    xlabel('Species')
    pause

    %plot population stats
    subplot(211)
    plot(T,[totalpoph,maxpoph],'LineWidth',2)
    legend('total population','biggest sp population')
    subplot(212)
    plot(T,[biodivhistory,sphistory],'LineWidth',2)
    legend('biodiversity','number of species')
    pause

    %plot structure analysis
    subplot(211)
    plot(T,str(:,2)','r-',T,str(:,4)','g-',T,str(:,6)','y-')
    legend('keystones','weeds','canaries')
    subplot(212)
    plot(T,str(:,1)','r-',T,(str(:,3))','g-',T,str(:,5)','y-')
    legend('keystones','weeds','canaries')
    pause
end
if strcmp(displayr,'compact') || strcmp(displayr,'complete')
    %display brief summary :
    summ=display_end(biodivhistory,lifespan,totalpoph,str);
end

%Save data : ----------------------------------------------------------------
if reverserows==1
    vars2save{1}=invrows(biodivhistory(firstDsave:end,:));
    vars2save{2}=invrows(preshistory(firstDsave:end,:));
    vars2save{3}=invrows(pophistory(firstDsave:end,:));
    vars2save{4}=lifespan(1,:);
    vars2save{5}=invrows(str(firstDsave:end,:));
    vars2save{6}=invrows(fluxhistory(firstDsave:end,:));
else
    vars2save{1}=biodivhistory(firstDsave:end,:);
```

```
    vars2save{2}=preshistory(firstDsave:end,:);
    vars2save{3}=pophistory(firstDsave:end,:);
    vars2save{4}=lifespan(1,:);
    vars2save{5}=str(firstDsave:end,:);
    vars2save{6}=fluxhistory(firstDsave:end,:);
end

if reverserows==1
    row_nb=(time:-1:firstDsave);
else
    row_nb=(firstDsave:time);
end

if strcmp(saveopt,'auto')   %automatic save
    w2save=2;               %minimum (1) by default, 2 for complete save
    dmm=fix(clock);
    date=[num2str(dmm(2)),'-',num2str(dmm(3)),'-',num2str(dmm(4)),...
        '-',num2str(dmm(5)),'-',num2str(dmm(6))];
    info=['K',num2str(K),'t',num2str(t)];
    name=[date,info];
elseif strcmp(saveopt,'manual')   %manual save (default)
    w2save=input('Save results ? No : 0, Yes : 1,  Complete : 2. ');
    if w2save≠0
        name=input('Enter the name of your file.');
        while ¬isa(name,'char')
            name=input('Enter the ''name'' of your file.');
        end
    end
else
    w2save=0;
end

if (w2save==1) || (w2save==2)
    save_file(vars2save{1},[name,'_Hill-biodiv.txt'],row_nb)
    save_file(vars2save{2},[name,'_pres-absc.txt'],row_nb)
end
if w2save==2    %Complete save include save of lifespan, structure analisys
    %and influx-outflux
    save_file(vars2save{3},[name,'_population.txt'],row_nb)
    save_file(vars2save{4},[name,'_lifespan.txt'])
    save_file(vars2save{5},[name,'_struct.txt'],row_nb)
    save_file(vars2save{6},[name,'_flux.txt'],row_nb)
    savegroup(Ks,Wd,Cn,[name,'_groups.txt'])
    savevar=[name,'_vars.mat'];
    save(savevar)
end

%_________________________________________________________________________


%Program designed by C lment VIGUIER (Polytech'Nice Sophia - GB)
%under James DYKE's direction (University of Southampton - ECS)
%with collaboration of Patrick DONCASTER (University of Southampton)
```

```
%SCRIPT OF DATA SET BUILDING AND ANALYSIS FOR ECOSYSTEM MODEL

%Before running this script be aware of the time used by the simulation to
%provide rresults. The Matlab profiler tool can give you a good idea of
%time necessary for particular parameters values.

%Parameters you can test :
%-

%Notice that N can't be change in this version

clc
clear all
format compact

tic

%set number of iteration and parameters sets
nbrun=40;

%list of the parameters you want to test, has to be consistent with
%parameters values contained in variable 'val'

%each row is a set of parameters
mode=['0d';'0d';'0d';'0d';'0b';'0b';'0b';'0b';'0b';'0b';...
    'Dd';'Dd';'Dd';'Dd';'Db';'Db';'Db';'Db';'Db';'Db';...
    'P0';'P0';'P0';'P0';'P0';'PD';'PD';'PD';'PD';'PD'];
val=[0,0.06;0,0.09;0,0.1;0,0.12;0,-10;0,-14;0,-16;0,-18;0,-22;0,-30;...
    1,0.06;1,0.09;1,0.1;1,0.12;1,-10;1,-14;1,-16;1,-18;1,-22;1,-30;...
    0,0;0.4,0;0.5,0;0.6,0;1,0;0,1;0.4,1;0.5,1;0.6,1;1,1];


%CHANGE THE SYSTEM OF CALLING THE MODEL TO MATCH WITH FORMAT OF INPUT
%ARGUMENTS


nbset=size(mode,1);
if nbset==0
    nbset=1;    %will use the default parameter set
end

%run simulations
for i=1:nbset
    disp ('Simulation is running')
    clear vars data data2
    data=zeros(nbrun,167);
    if size(mode,1)≠0
        for r=1:nbrun
            data(r,1:167)=ecosystem_a(mode(i,:),val(i,:));
        end
    else
        for r=1:nbrun
            data(r,1:167)=ecosystem_a();
        end
    end
    D=data(:,1:17);
    data2=[mean(D,1);std(D)];
    biod(1:2,i)=data2(:,1);
    mtotal(1:2,i)=data2(:,2);
    finaltotal(1:2,i)=data2(:,10);
    mspecies(1:2,i)=data2(:,3);
    finalspecies(1:2,i)=data2(:,11);
```

```
    canariesnb(1:2,i)=data2(:,8);
    canariespop(1:2,i)=data2(:,9);
    weedsnb(1:2,i)=data2(:,6);
    weedspop(1:2,i)=data2(:,7);
    keystonesnb(1:2,i)=data2(:,4);
    keystonespop(1:2,i)=data2(:,5);
    endcanariesnb(1:2,i)=data2(:,16);
    endcanariespop(1:2,i)=data2(:,17);
    endweedsnb(1:2,i)=data2(:,14);
    endweedspop(1:2,i)=data2(:,15);
    endkeystonesnb(1:2,i)=data2(:,12);
    endkeystonespop(1:2,i)=data2(:,13);

    %display species abundance distribution for each parameter tested
    pop(i)={data(:,18:end)};
    %SAD(pop{i},1000)
end

dmm=fix(clock);
date=[num2str(dmm(2)),'-',num2str(dmm(3)),'-',num2str(dmm(4)),...
    '-',num2str(dmm(5))];
info=['nbsim',num2str(i),'nbrun',num2str(nbrun)];
name=[date,info,'_vars.mat'];
save(name)
disp(['All variables have been saved in file :',name])

toc
```

### A.2.2 Functions

In this section you will fin the main functions used is the program.

```
function h=biodiv(mat)
%Calculation of Hill biodiversity : ________________________________________
mat=sqrt(mat);
s=1./sum(mat,2);
b=bsxfun(@times,mat.^2,s.^2);
h=1./sum(b,2);
%__________________________________________________________________________
```

```
function win=compet(list,mode,coeff,phi)
%Drive the tournament between individuals in the list :
%first column is the aim of the fight
%second column is the id/index of the finger (not necesarely unique)

%This parameter determines the strenght of the competition, 0 leads to a
%completely deterministiccompetition, 0.5 to a compeition based on
%probabilities equal to rd and 1 to a completely random competition

%non zero rows are group by patch claimed
list=list(any(list,2),:);
list=sortrows(list,1);

l=size(list,1);
i=1;
win=zeros(l,2);

while i<l
    if list(i,1)==list(i+1,1)
        s1=list(i,2);
        s2=list(i+1,2);
        %figth threshold depends of competition mode/stage
        if mode==2
            rd=1-coeff(s1,s2);
        else
            rd=coeff(s2,s1)/(coeff(s2,s1)+coeff(s1,s2));
        end
        %probability of victory is depending on phi value and rd value
        rd2=rd*(1-phi)+phi*rand;
        if 0.5<rd2
            %agent of species s1 win this round
            list(i+1,:)=[];
        else
            %agent of species s2 win this round
            i=i+1;
        end
    else
        %the agent win the stage
        win(i,:)=list(i,:);
        i=i+1;
    end
    l=size(list,1);
end

%list of winners
win(i,:)=list(i,:);


win=win(any(win,2),:);
```

```
function s=invasion
%Pick a species in absent specie%, update presence/absence array

global absc
global pres
global sp

if sum(absc)≠0
    list=sp(absc);                    %list of species absent in environment
    r=round(1+(length(list)−1)*rand);
    s=list(r);                        %pick randomly a species in this list
    pres(s)=1;
    absc(s)=0;
else
    s=0;
end
```

```
function save_file(mat,name,num_row,num_col)
%save mat in file called 'name' and index columns and rows if there i many
fclose('all');

[n,m]=size(mat);

switch nargin
    case 1
        name=num2str(fix(clock));
    case 2
        num_col=(1:m);
        num_row=(1:n)';
    case 3
        num_col=(1:m);
    case 4
    otherwise
        disp('Too many input arguments.')
end

if size(num_col,2)≠m
    num_col=(1:m);
end
if size(num_row,1)==1
    num_row=num_row';
end
if size(num_row,1)≠n
    num_row=(1:n)';
end

if m>1 && n>1
    if ischar(num_row)
        format=['%c\t',repmat('%3g\t',1,m),'\n'];
    else
        format=['%g\t',repmat('%3g\t',1,m),'\n'];
    end
    if ischar(num_col)
        f_line=['\t ',repmat('%c\t',1,m),'\n'];
    else
        f_line=['\t ',repmat('%g\t',1,m),'\n'];
    end
    mat=[num_row,mat]';
    fid=fopen(name,'wt');
    fprintf(fid,f_line,num_col);
    fprintf(fid,format,mat);
    fclose(fid);
elseif m>1
    if ischar(num_col)
        f_line=[repmat('%c\t',1,m),'\n'];
    else
        f_line=[repmat('%g\t',1,m),'\n'];
    end
    format=[repmat('%3g\t',1,m),'\n'];
    fid=fopen(name,'wt');
    fprintf(fid,f_line,num_col);
    fprintf(fid,format,mat);
    fclose(fid);
elseif n>1
    if ischar(num_row)
        format='%c\t%3g\t\n';
    else
        format='%g\t%3g\t\n';
    end
```

```
    format='%g\t%3g\t\n';
    mat=[num_row,mat]';
    fid=fopen(name,'wt');
    fprintf(fid,format,mat);
    fclose(fid);
else
    disp('File too small to be saved.')
end

fclose('all');
```

### A.2.3 Variables

This is not a exhaustive list of all variables, but it gives you an idea of logic I tried to keep in variables' name.

*This part is not yet available. I will do the necessary to complete it as soon as possible. Excuse me for this lack.*
