# Supplementary material for: Early warning of critical transitions in biodiversity from compositional disorder
Source: Ecology. 2016 Nov 3;97(11):3079–90. doi: 10.1002/ecy.1558 (PMC6849621; doi:10.1002/ecy.1558)
Supplement: Supplementary file 5 [file ECY-97-3079-s005.pdf]

Doncaster, C. P., C. Viguier, and J. G. Dyke. 2016. Early warning of a critical transitions in biodiversity from compositional disorder. *Ecology*

Supplement

Description of source code for Agent Based Model.

Author(s)

C. P. Doncaster
Biological Sciences
University of Southampton
Southampton SO17 1BJ, UK
cpd@soton.ac.uk

C. Viguier
University of Nice Polytech Nice-Sophia
06903 Sophia-Antipolis Cedex, France.
viguier@polytech.unice.fr

J. G. Dyke
Geography and Environment
University of Southampton
Southampton SO17 1BJ, UK
J.Dyke@soton.ac.uk

File list (files found within DataS1.zip)

```
biodiv.m
clean.m
compet.m
create_com.m
display_end.m
display_start.m
ecosystem_a.m
flux.m
flux2ph.m
fluxmeasure.m
inv_norm.m
invasion.m
```

```
invrows.m
mapping.m
newprop.m
plotsurf.m
Repeat_simulation.m
rescaling01.m
SAD.m
save_file.m
savegroup.m
set_parameters.asv
set_parameters.m
short_manual.txt
srand.m
structure.m
temp_biod.m
tradeofalpha.m
tradeofinv.m
tradeofsig.m
urand.m
VIGUIER_program-user-guide.pdf
```

Description

The simulation results presented in the main text and Appendix S1 was produced with the code "Ecosystem Simulation Version 2". The entire source code is available for download in DataS1.zip. The archive consists of 29 individual Matlab function files, a parameter setting asv file, a text-file short manual, and a PDF user manual.

The individual Matlab function files are called from Repeat_simulation.m which serves as a wrapper for all functions and is the file that is executed in order to produce simulation results.

Repeat_simulation.m

The script Reapeat_simulation allows the simulation to be run with the same community (alpha and c, imported or created at the beginning of the session) or different community (alpha matrix and c created for every simulation).

The source code for this script contains extensive comments.

The vectors "mode" contains on every row the parameters for one set of simulations, and the vector "value" contains all values of these parameters.

-- PARAMETERS --

K - size of the environment (~1000)

D - type of degradation (0:none, 1:1 fluctuating phase,2: fluctuation around ddef then fluctuation and global increase,3: linear increase and decrease)

d - default value of death rate (~0.1)

c - minimum value of intrinsic growth rate (~0.1>d)

n - number of starting species (<K/30)

i - invasion flux, 0: constant, 1: fluctuating

t - number of time-steps (~1000)

s - type of trade-off, 'linear' or 'sigmoidal' (default)

b - slope of sigmoidal trade-off (~-16)

P - randomness of competition (0<P<1)

f - detection threshold (~5)

It is possible to define alternative settings by modifying the default value of all variables (not only variables listed hereabove) in the function "ecosystem_a". For example changing the total number of species N need to change the value of N in the function "ecosystem_a" (in this case, be sure that the number of species defined in alpha and c are consistent if you use the same community for all simulations).

- LOAD ALPHA AND C --

In order to load custom ALPHA and C, two separate files are required. Enter the name (or path and name) of these files when the script ask you to do so.

Note: the Number of species defined in alpha and c, has to be consistent with the value of N in the function "ecosystem_a"

-- USE THE SAME ALPHA AND C

It is possible to use the same alpha and c for all simulations, in this case you have the possibility to save these variables at the end of session.

You can change the number of species is the community, and the value of beta for the trade-off in the script Repeat_simulation.

-- SAVING AND DISPLAY OPTION –

Option specific from "ecosystem_a" saving and displaying have to be mofied directly in the code of the function.

Below are the default values of these options:

saveopt='none';	%'auto' or 'manual' save (or 'none') auto' gives an automatic name and saves biodiversity and presence matrix.
displayr='complete';	%display results : 'complete', 'compact' or 'none' complete shows all diagrams, compact is limited to summaries.
firstDsave=600;	%day of beginning of saving (stabilization phase finished around 600)
reverserows=1;	%reverse order of rows (=1) or not (=0)
